# Correction: *A randomised controlled trial of a facilitated home-based rehabilitation intervention in patients with heart failure with preserved ejection fraction and their caregivers: the REACH-HFpEF Pilot Study*

**DOI:** 10.1136/bmjopen-2017-019649corr1

**Published:** 2019-03-23

Lang CC, Smith K, Wingham J, *et al*. A randomised controlled trial of a facilitated home-based rehabilitation intervention in patients with heart failure with preserved ejection fraction and their caregivers: the REACH-HFpEF Pilot Study. *BMJ Open* 2018;8:e019649.doi: 10.1136/bmjopen-2017-019649

An error has been identified in the reported results of fidelity scores, which should have been reported as median rather than mean scores. The correct supplementary e Table 4 is shown below. Additionally, Joanne Coyle and not Karen Coyle reviewed the fidelity of the intervention delivery. These changes to the manuscript do not impact on the overall conclusions of the manuscript.

Supplementary e Table 4. Fidelity of Intervention delivery

**Table:** 

	Item 1 Involve- ment	Item 2 Assess- ment	Item 3 Plan	Item 4 Under- stadning	Item 5a Support- why to change	Item 5b Progress review	Item 6 Physical activity	Item 7 Emotion	Item 8 Medic- ation	Item 9 Care giver involve- ment	It em 10 care -giver emotion	Item 11 care- giver well being	Item 12 closure
N patients	6	6	6	6	6	6	6	6	6	6	6	6	6
Median score	3.5	4.0	3.4	4.5	3.50	3.5	3.8	3.8	4.5	3.0	2.5	2.0	2.9

