# Supplementary material for: A randomised controlled trial of a facilitated home-based rehabilitation intervention in patients with heart failure with preserved ejection fraction and their caregivers: the REACH-HFpEF Pilot Study
Source: BMJ Open. 2018 Apr 9;8(4):e019649. doi: 10.1136/bmjopen-2017-019649 (PMC5893929; doi:10.1136/bmjopen-2017-019649)
Supplement: Supplementary file 1 [file bmjopen-2017-019649supp001.pdf]

eTable1. Unit costs

**Resource use/Item**	**Unit cost 2016 £**	**Source**
**Primary Care cost per visit/appointment**		
GP (surgery)	£31.00	Curtis and Burns, 2016
GP (home)	£74.98	Curtis and Burns, 2015
GP (phone)	£22.29	Curtis and Burns, 2015
Practice nurse (surgery)	£11.11	Curtis and Burns, 2016, Curtis and Burns, 2015
Practice nurse (home)	£18.80	Curtis and Burns, 2016, Curtis and Burns, 2015, Curtis, 2010.
Practice nurse (phone)	£4.30	Curtis and Burns, 2016, Curtis and Burns, 2015
Heart failure nurse	£22.11	Curtis and Burns, 2016,
Physiotherapist	£77.52	Curtis and Burns, 2016, Curtis, 2010.
Occupational therapist	£71.40	Curtis and Burns, 2016, Curtis, 2010.
Community/district nurse	£39.51	Curtis and Burns, 2016, Curtis, 2010.
Health visitor	£27.22	Curtis and Burns, 2015, Curtis, 2010.
Other primary/community service	£22.11	Curtis and Burns, 2016,
**Secondary care cost per event**		
Hospital admission (HF)	£4,668.66	Department of Health, 2016
Hospital admission (non-HF)	£3,966.57	Zannad *et al.* 2011, Department of Health, 2016
Hospital admission (overall)	£4,282.51	Combination of HF and non-HF admission cost, weighted according to admissions recorded in pilot
A&E attendance	£137.82	Department of Health, 2016
Day hospital attendance	£319.33	Department of Health, 2016
Outpatient cardiology appointment	£135.68	Department of Health, 2016
Outpatient cardiac or HF nurse	£102.96	Department of Health, 2016
Other outpatient appointment	£116.54	Department of Health, 2016

**Social & community care visits:**		
Social worker	£79.00	Curtis and Burns, 2016
Home care /home help	£12.00	Curtis and Burns, 2016
Voluntary agency	£10.00	Curtis and Burns, 2016
Day care	£46.00	Curtis and Burns, 2016
Drop in club	£13.00	Curtis and Burns, 2016
**Medications** (estimated 6-month cost per person)		
Angiotension 2-receptor antagonist	£15.09	Joint Formulary Committee 2017, OpenPrescribing.net
ACE inhibitor	£6.92	Joint Formulary Committee 2017, OpenPrescribing.net
Aldosterone receptor antagonist	£63.05	Joint Formulary Committee 2017, OpenPrescribing.net
Anti-coagulant	£8.34	Joint Formulary Committee 2017, OpenPrescribing.net
Beta-blocker	£6.15	Joint Formulary Committee 2017, OpenPrescribing.net
Digoxin	£18.00	Joint Formulary Committee 2017, OpenPrescribing.net
Ivabradine	£258.24	Joint Formulary Committee 2017, OpenPrescribing.net
Loop diuretic	£7.96	Joint Formulary Committee 2017, OpenPrescribing.net
Nitrate + hydralazine	£589.60	Joint Formulary Committee 2017, OpenPrescribing.net
Thiazide diuretic	£9.61	Joint Formulary Committee 2017, OpenPrescribing.net
**Patient & Caregiver Time** cost per unit		
Caregiver time, hour	£24.00	Curtis and Burns, 2016
Non-caregiver time, hour	£24.00	Curtis and Burns, 2016
Caregiver time off work, per day	£122.31	HM Revenue & Customs, 2017
Non-caregiver time off work, per day	£122.31	
Patient time off work, per day	£96.15	HM Revenue & Customs, 2017

References

Curtis L, Burns A. *Unit Costs of Health & Social Care 2016*. Personal Social Services Research Unit, The University of Kent. 2016.

Curtis L, Burns A. *Unit Costs of Health & Social Care 2015*. Personal Social Services Research Unit, The University of Kent. 2015.

Curtis L. *Unit Costs of Health & Social Care 2010*. Personal Social Services Research Unit, The University of Kent. 2010.

Department of Health, *NHS reference costs* 2015 to 2016, 2016.

HM Revenue & Customs, *Distribution of median and mean income and tax by age range and gender*: 2014 to 2015, UK National Statistics. 2017

BNF. Joint Formulary Committee. *British National Formulary* (online) London: BMJ Group and Pharmaceutical Press <http://www.medicinescomplete.com> [Accessed on 22.06.2017]

eTable 2. Patient and caregiver acceptability with trial participation at 6-months follow up

**What was your overall impression of taking part in the study?**	**Very good or good** N (%)	**Acceptable** N (%)	**Poor or very Poor** N (%)
**Patients**			
Intervention group, N = 21	19 (90)	2 (10)	0 (0)
Control group, N = 23	23 (100)	0 (0)	0 (0)
Both groups, N =44	42 (95)	2 (5)	0 (0)
**Caregivers**			
Intervention group, N = 8	7 (88)	1 (12)	0 (0)
Control group, N = 9	5 (55)	4 (45)	0 (0)
Both groups, N = 17	13 (72)	5 (28)	0 (0)

eTable 3. Patients and caregivers acceptability of REACH-HF intervention

The following are verbatim quotes of the positive experiences of the REACH-HF from patients and their caregivers.

Patient: *"I felt like giving her [the REACH-HF intervention facilitator] a hug to say thanks…you don't know what you've done for me… reach doesn't know what they have done for me" …. "Yeh so if anyone is listening to this and I hope 'youse' are and you are wanting to go on this programme, please go on it!!"*

Exercise

Caregiver: *"Yes, it was very helpful [exercise programme]. It really was. Helpful for me, as I say cos I started going out walking. We did the exercises…I'd never seen (my husband) and I laugh so much doing the exercises. You know, we had great fun. And the lady's straight face and…he would…he would put on funny poses and we laughed and we laughed. We thought…you know, we haven't laughed like that for a long, long time, you know. And it was really good. It really was."*

Patient: *After I think it was 9 weeks every single day I was trying my damdest to get past this, but I could not get past the (chair based exercise) warm up thing, so I said the facilitator I'm going to have to stop this (exercise) …. And she went 'no if you can't do that what do you love doing?' I says I love walking so she said 'right if you want to go out for a walk lets go out for a walk'…'*

Role of facilitator (education, support and reassurance)

Patient: *"I think that…reading the manual, talking to 'the nurse ', was very helpful for me in so many different ways. Helping me to understand heart failure….she encouraged me to go out walking…. Just the reassurance that things were better, that there was somebody there that was willing to, erm, say, well, okay, you're doing well. Even just the smallest amount of encouragement. And 'my husband' always felt better after the facilitator went away. Because she felt…almost like a little security blanket, if you want to say. That somebody was there, somebody was asking."*

Facilitator as motivator

Patient: *'she was wonderful, encouraged me to do more walking and so on and I knew I could do it '*

Supporting behavioural change

Patient: *I tried to watch what I am eating more, my diet I take far more care …. I'm eating a lot more fish and vegetables rather than meat .*

Emotional support for patents and caregivers

Patient: "I'd pulled myself in I was really very inward and they were all saying you should go out with your friends ,or do this, or have them up…..I think being able to speak about it was helpful because that's not me."

Caregiver: *"What I've found about this Programme was….the nurse that came. You could talk it through. After talking to her, I didn't have quite so bad a fear of it [heart failure]. You could tell her how frightened you were,..it's nice to have someone professional to say, well, look, okay, that's that day. I didn't actually realise that until she came, how good it was to actually sit and openly speak about it and openly say, well, ask advice and things. It was lovely having her. You know, it was just a support."*

Caregiver: "I think maybe it's helped him think I can live with this … you know it's not – it doesn't mean the end of things"

eTable 4. Fidelity of intervention delivery

	Item 1 Involve-ment	Item 2 Assess-ment	Item 3 Plan	Item 4 Under-stand	Item 5a Support – why to change	Item 5b Review	Item 6 Physical activity	Item 7 Emotion	Item 8 Medic-ation	Item 9 Care-giver	Item 10 Care-giver emotion	Item 11 Care-giver well-being	Item 12 Closure
**N patients**	6	6	6	6	6	6	6	6	6	6	6	6	6
**Mean score**	3.3	4.0	3.7	4.5	3.2	3.4	4.5	5.5	5.0	2.5	4.5	2.1	4.0

eTable 5. Within group difference in patient outcomes between baseline, and 4- and 6-month follow-up

	4-month follow-up vs. baseline<br>Within group mean difference (95% CI)		6-month follow-up vs. baseline<br>Within group mean difference (95% CI)	
	**Intervention**	**Control**	**Intervention**	**Control**
Primary outcome				
MLHFQ, Overall	-2.0 (-9.2 to 5.2)	3.0 (-4.7 to 10.7)	-8.3 (-16.8 to 0.1)	3.9 (-4.9 to 12.6)
MLHFQ, Physical	-1.1 (-4.5 to 2.3)	2.0 (-1.8 to 5.9)	-3.3 (-7.3 to 0.7)	1.6 (-2.7 to 5.9)
MLHFQ, Emotional	-0.6 (-2.7 to 1.5)	1.5 (-1.1 to 4.1)	-1.6 (-3.9to 0.7)	1.3 (-1.5 to 4.2)
Secondary outcomes				
HADS, Anxiety	0.0 (-1.4 to 1.3)	0.7 (-0.8 to 2.1)	0.1 (-1.5 to 1.8)	0.3 (-1.6 to 2.2)
HADS, Depression	-0.4 (-1.7 to 0.8)	1.0 (0.1 to 2.0)	-0.2 (-1.2 to 0.7)	1.3 (-0.2 to 2.8)
Heart-QoL, Global	0.0 (-0.2 to 0.3)	-0.2 (-0.5 to 0.0)	0.3 (0.0 to 0.5)	-0.2 (-0.6 to 0.1)
Heart-QoL, Physical	0.1 (-0.2 to 0.3)	-0.2 (-0.5 to 0.0)	0.3 (0.0 to 0.6)	-0.2 (-0.6 to 0.1)
Heart-QoL, Emotional	0.0 (-0.3 to 0.2)	-0.2 (-0.5 to 0.2)	0.1 (-0.1 to 0.4)	-0.2 (-0.6 to 0.2)
EQ-5D-3L, utility score	0.01 (-0.1 to 0.12)	-0.06 (-0.12 to -0.01)	0.05 (-0.08 to 0.18)	-0.03 (-0.12 to 0.07)
SCHFI, Maintenance	15.5 (9.4 to 21.5)	5.8 (1.1 to 10.6)	9.8 (4.5 to 14.8)	5.1 (-1.5 to 11.8)
SCHFI, Management	12.1 (1.3 to 22.9)	-5.4 (-14.9 to 4.2)	8.6 (-4.4 to 21.6)	-1.0 (-14.5 to 12.5)
SCHFI, Confidence	3.5 (-10.0 to 17.0)	-7.0 (-15.4 to 1.4)	0.2 (-10.4 to 10.8)	-3.1 (-15.3 to 9.0)
ISWT (metres)	5.0 (-27.9 to 37.9)	-12.9 (-41.3 to 15.4)	-7.9 (-44.6 to 28.7)	4.1 (-17.3 to 25.5)
Accelerometry, average time/day at ≤ 20mg	-9 (-36 to 18)	26 (5 to 48)	8 (-14 to 30)	26 (-5 to 60)

Accelerometry, average time/day at 21 to 40mg	11 (1 to 20)	-13 (-22 to -3)	5 (-3 to 13)	-11 (-25 to 2)
Accelerometry, average time/day at 41 to 60mg	2 (-6 to 10)	-6 (-12 to 0)	-2 (-8 to 5)	-7 (-17 to 3)
Accelerometry, average time/day at 61 to 80mg	0 (-5 to 6)	-3 (-7 to 1)	-4 (-10 to 2)	-4 (-10 to 2)
Accelerometry, average time/day at 81 to 100mg	0 (-3 to 3)	-1 (-4 to 1)	-2 (-5 to 1)	-1 (-5 to 2)
Accelerometry, average time/day at > 100mg	-4 (-9 to 1)	-3 (-6 to 0)	-5 (-11 to 1)	-4 (-8 to 1)

eTable 6. Within group difference in caregiver outcomes between baseline, and 4- and 6-month follow-up

	4-month follow-up vs. baseline<br>Within group mean difference (95% CI)		6-month follow-up vs. baseline<br>Within group mean difference (95% CI)	
	**Intervention**	**Control**	**Intervention**	**Control**
HADS, Anxiety	-2.1 (-5.4 to 1.1)	0.6 (-2.2 to 3.4)	-3.0 (-5.5 to -0.5)	0.9 (-1.5 to 3.3)
HADS, Depression	0.3 (-2.2 to 2.7)	0.7 (-1.6 to 3.0)	-0.8 (-2.6 to 1.1)	0.9 (-2.2 to 4.0)
FAMQOL, Overall	-3.0 (-10.4 to 4.4)	-2.6 (-6.7 to 1.5)	-6.3 (-13.1 to 0.6)	-1.9 (-7.8 to 3.9)
FAMQOL, Physical	-1.8 (-4.1 to 0.6)	0.0 (-2.3 to 2.3)	-1.9 (-3.3 to -0.4)	0.6 (-0.6 to 1.7)
FAMQOL, Psychological	-0.6 (-3.0 to 1.8)	-1.5 (-3.8 to 0.8)	-1.1 (-3.7 to 1.5)	-1.2 (-4.0 to 1.6)
FAMQOL, Social	-1.4 (-3.7 to 1.0)	-1.0 (-3.5 to 1.5)	-2.0 (-3.4 to -0.6)	-0.6 (-3.1 to 2.0)
EQ5D-3L, utility score	0.01 (-0.05 to 0.07)	0.01 (-0.10 to 0.12)	-0.03 (-0.12 to 0.07)	-0.08 (-0.19 to 0.02)
CBQ-HF, Physical	-2.4 (-5.6 to 0.8)	2.6 (-0.7 to 5.9)	0.0 (-2.4 to 2.4)	1.4 (-0.1 to 3.0)
CBQ-HF, Emotional	-1.1 (-5.0 to 2.8)	1.5 (-3.1 to 6.1)	-0.9 (-5.1 to 3.4)	4.0 (-2.4 to 10.4)
CBQ-HF, Social Life	-0.1 (-0.4 to 0.2)	0.2 (-0.8 to 1.2)	0.1 (-0.4 to 0.7)	0.6 (-1.1 to 2.2)
CBQ-HF, Lifestyle	0.8 (-1.2 to 2.7)	0.1 (-1.5 to 1.7)	0.6 (-1.4 to 2.7)	2.0 (-0.4 to 4.4)
CC-SCHFI, Maintenance	13.8 (-6.0 to 33.5)	1.4 (-5.6 to 8.4)	15.9 (-2.9 to 34.6)	11.9 (0.6 to 23.1)
CC-SCHFI, Management	5.0 (-10.1 to 20.1)	7.5 (-11.5 to 26.5)	5.0 (-27.9 to 37.9)	-1.4 (-19.3 to 16.4)

CC-SCHFI, Confidence	2.1 (-13.4 to 17.6)	-9.6 (-23.7 to 4.6)	4.9 (-11.1 to 20.8)	5.4 (-10.9 to 21.8)

Table e7. Wider healthcare and societal utilisation at 6-months follow up

	Intervention		Control	
	Appointments/ visits per person	Cost £ per person	Appointments/ visits per person	Cost £ per person
	mean (SD) N	mean (SD)	mean (SD) N	mean (SD)
**Primary Care Appointments**				
GP (surgery)	5.36 (7.68) 22	£166.16 (£238.08)	2.78 (2.04) 23	£86.18 (£63.24)
GP (home)	0.45 (0.91) 22	£33.74 (£68.24)	0.61 (2.29) 23	£45.74 (£171.71)
GP (phone)	0.64 (1.29) 22	£14.27 (£28.76)	0.91 (3.36) 23	£20.29 (£74.90)
Practice nurse (surgery)	2.77 (2.69) 22	£30.77 (£29.88)	2.61 (2.52) 23	£28.99 (£27.99)
Practice nurse (home)	0.09 (0.43) 22	£1.69 (£8.08)	0.00 (0.00) 23	£0.00 (£0.00)
Practice nurse (phone)	0.27 (0.94) 22	£1.16 (£4.04)	0.39 (1.88) 23	£1.68 (£8.08)
Heart failure nurse	0.00 (0.00) 22	£0.00 (£0.00)	0.00 (0.00) 23	£0.00 (£0.00)
Physiotherapist	2.73 (12.79) 22	£211.62 (£984.45)	1.00 (3.80) 23	£77.52 (£294.56)
Occupational therapist	0.00 (0.00) 22	£0.00 (£0.00)	0.52 (2.50) 23	£39.05 (£187.74)
Community/district nurse	0.05 (0.21) 22	£1.98 (£8.30)	0.39 (1.88) 23	£15.41 (£74.27)
Health visitor	0.00 (0.00) 22	£0.00 (£0.00)	0.00 (0.00) 23	£0.00 (£0.00)
Primary Care Total	12.36 (17.84) 22	£461	9.22 (11.10) 23	£315
**Secondary care**				
Hospital admission	0.18 (0.50) 22	£770.85 (£2141.25)	0.30 (0.63) 23	£1284.75 (£2697.98)
A&E attendance	0.00 (0.00) 22	£0.00 (£0.00)	0.09 (0.29) 23	£12.40 (£39.97)

Day hospital attendance	0.32 (0.72) 22	£102.18 (£229.92)	0.04 (0.21) 23	£12.77 (£67.06)
Outpatient cardiology appointment	0.41 (0.67) 2	£55.63 (£90.90)	0.57 (1.08) 23	£77.34 (£146.53)
Outpatient cardiac or HF nurse	0.05 (0.21) 22	£5.15 (£21.62)	0.00 (0.00) 23	£0.00 (£0.00)
Other outpatient appointment	0.00 (0.00) 22	£0.00 (£0.00)	0.00 (0.00) 23	£0.00 (£0.00)
Secondary Care Total	0.95 (1.00) 22	£934	1.00 (1.48) 23	£1,387
Social worker	0.45 (1.41) 22	£35.55 (£111.39)	0.00 (0.00) 23	£0.00 (£0.00)
Home care /home help	4.41 (20.68) 22	£52.92 (£247.20)	3.48 (11.01) 23	£41.76 (£132.00)
Day care	0.00 (0.00) 22	£0.00 (£0.00)	6.26 (20.74) 23	£287.96 (£952.20)
Drop in club	0.00 (0.00) 22	£0.00 (£0.00)	0.00 (0.00) 23	£0.00 (£0.00)
Other day care service	0.00 (0.00) 22	£0.00 (£0.00)	0.00 (0.00) 23	£0.00 (£0.00)
Social Care Total	4.86 (20.85) 22	£88	9.74 (22.49) 23	£330
Voluntary agency visit	0.00 (0.00) 22	£0.00 (£0.00)	0.09 (0.42) 23	£0.90 (£4.20)
Other primary or community based service	0.00 (0.00) 22	£0.00 (£0.00)	0.16 (0.80) 23	£3.54 (£17.69)
All Health & Social Care Visits Total	18.18	£1,484	20.20	£2,036
	% prescribed	Cost per person	% prescribed	Cost per person

	Mean, N	mean	Mean, N	mean
**Medications**				
Angiotensin II receptor antagonist	29% 25	£4.38	28%, 25	£4.23
ACE inhibitor	44%, 25	£3.04	48%, 25	£3.31
Aldosterone receptor antangonist	16%, 25	£10.09	24%, 25	£15.13
Anti-coagulant	15%, 25	£1.25	53%, 25	£4.42
Beta-blocker	56%, 25	£3.43	44%, 25	£2.69
Digoxin	8%., 25	£1.44	12%, 25	£2.16
Ivabradine	4%, 25	£10.33	4%, 25	£10.33
Loop diuretic	77%, 25	£6.14	76%, 25	£6.06
Nitrate	39%, 25	£108.15	19%, 25	£52.69
Thiazide diuretic	5%, 25	£0.48	1%, 25	£0.10
All Medications Total		£149		£101
All Health & Social Care Total		£1,632		£2,137
**Informal care**				
Caregiver hours per week	3.03 (5.86) 22	£72.72 (£140.64)	12.41 (30.30) 23	£297.60 (£727.20)

Non-caregiver hours per week	4.98 (12.57) 22	£119.52 (£301.68)	0.46 (1.31) 23	£11.04 (£31.44)
Total caring hours per week	8.01	£192	12.86	£309
Total caring hours per 6 months	208	£4,998.24	334	£8,025
Caregiver days off work	0.14 (0.64) 22	£17.12 (£78.28)	1.00 (4.38) 23	£122.31 (£535.71)
Non-caregiver days off work	0.14 (0.64) 22	17.12 (£78.28)	0.00 (0.00) 13	£0.00 (£0.00)
Total days off work (6-mths)	0.28	£34.25	1	£122.31
Patient days off work	0.00 (0.00) 22	£0.00 (£0.00)	0.00 (0.00) 23	£0.00 (£0.00)
Informal Care Total		£5,032		£8,147
**All Health, Informal & Social Care Total**		**£6,665**		**£10,284**
