# Supplementary material for: A randomised controlled trial of a facilitated home-based rehabilitation intervention in patients with heart failure with preserved ejection fraction and their caregivers: the REACH-HFpEF Pilot Study
Source: BMJ Open. 2018 Apr 9;8(4):e019649. doi: 10.1136/bmjopen-2017-019649 (PMC5893929; doi:10.1136/bmjopen-2017-019649)
Supplement: Author's manuscript [file bmjopen-2017-019649.draft_revisions.pdf]

BMJ Open

BMJ Open is committed to open peer review. As part of this commitment we make the peer review history of every article we publish publicly available.

When an article is published we post the peer reviewers' comments and the authors' responses online. We also post the versions of the paper that were used during peer review. These are the versions that the peer review comments apply to.

The versions of the paper that follow are the versions that were submitted during the peer review process. They are not the versions of record or the final published versions. They should not be cited or distributed as the published version of this manuscript.

BMJ Open is an open access journal and the full, final, typeset and author-corrected version of record of the manuscript is available on our site with no access controls, subscription charges or pay-per-view fees (http://bmjopen.bmj.com).

If you have any questions on BMJ Open's open peer review process please email info.bmjopen@bmj.com

BMJ Open

A Randomised Controlled Trial of a Facilitated Home-Based Rehabilitation Intervention in Patients with Heart Failure with Preserved Ejection Fraction and their Caregivers: REACH-HFpEF Pilot Study.

Journal:	*BMJ Open*
Manuscript ID	bmjopen-2017-019649
Article Type:	Research
Date Submitted by the Author:	22-Sep-2017

Complete List of Authors:	Lang, Chim C.; Univ Dundee, Division of Molecular & Clinical Medicine<br>Smith, Karen; University of Dundee, Molecular & Clinical Medicine/School of Nursing<br>Wingham, Jennifer; Royal Cornwall Hospitals NHS Trust, Research, Development and Innovation; University of Exeter Medical School,<br>Eyre, Victoria; Plymouth University, Peninsula Clinical Trials Unit<br>Greaves, Colin; University of Exeter Medical School, Institute of Health Research<br>Warren, Fiona; University of Exeter Medical School, Institute of Health Research<br>Green, Colin; University of Exeter Medical School, Institute of Health Research<br>Jolly, Kate; University of Birmingham<br>Davis, Russell; Sandwell & West Birmingham Hospitals NHS Trust, Dept of Cardiology<br>Doherty, Patrick Joseph; University of York, Health Science<br>Miles, Jackie; Anuerin Bevan University Health Board, Research and Development<br>Britten, Nicky; University of Exeter Medical School, PenCLAHRC: National Institute for Health Research (NIHR) Collaboration for Leadership in Applied Health Research and Care (CLAHRC) for the South West Peninsula; University of Exeter Medical School, Institute for Health Service Research<br>Abraham, Charles; University of Exeter Medical School, Psychology Applied to Health Group<br>Van Lingen, Robin; Royal Cornwall Hospitals NHS Trust, Cardiology<br>Singh, Sally; University Hospitals of Leicester NHS Trust, Cardiac/Pulmonary Rehabilitation<br>Paul, Kevin; Royal Cornwall Hospitals NHS Trust, REACH-HF Patient and Public Involvement Group, c/o Research, Development & Innovation<br>Hillsdon, Melvyn; University of Exeter<br>Sadler, Susannah; University of Exeter Medical School, Institute of Health Research<br>Hayward, Christopher; Plymouth University, Peninsula Clinical Trials Unit<br>Dalal, Hayes; Royal Cornwall Hospitals NHS Trust, Teaching and Research<br>Taylor, Rod; University of Exeter, Peninsula Medical School

Keywords:	Heart Failure,, Heart Failure with Preserved Ejection Fraction, Cardiac Rehabilitation, Randomised Controlled Trial, Complex Intervention

For peer review only

A Randomised Controlled Trial of a Facilitated Home-Based Rehabilitation Intervention in Patients with Heart Failure with Preserved Ejection Fraction and their Caregivers: REACH-HFpEF Pilot Study

Corresponding Author:

Chim Choy Lang,
Division of Clinical and Molecular Medicine
School of Medicine
Mail box 2
Ninewells Hospital & Medical School
Dundee
DD1 9SY
E: c.c.lang@dundee.ac.uk
T: 01382 383013

ISRCTN 57596739

Lang Chim C (Professor/Medical Consultant)[1], Smith Karen (Nurse Consultant)[1,2], Wingham Jenny (Honorary Clinical Senior Lecturer/Senior Clinical Researcher)[3,4], Eyre Victoria (Centre Manager)[5], Greaves Colin J (Associate Professor)[3], Warren Fiona C (Senior Lecturer)[3], Green Colin (Professor)[3], Jolly Kate (Professor) [6], Davis Russell C (Medical Consultant)[7], Doherty Patrick (Professor)[8], Austin Jackie (Lead Nurse Heart Failure/Cardiac Rehabilitation Research)[9], Britten Nicholas (Professor)[3], Abraham Charles (Professor)[3,] , Van Lingen Robin (Medical Consultant)[10], Singh Sally (Professor)[11], Paul Kevin (Patient Representative)[12], Hillsdon Melvyn (Professor)[13] , Sadler Susannah (Research Fellow)[3], Hayward Christopher (Senior Trial Manager)[14], Dalal Hayes M (General Practitioner/Honorary Clinical Associate Professor)[3,4], Taylor Rod S (Professor)[3] and on behalf of the REACH-HF investigators

[1]School of Medicine, University of Dundee, Ninewells Hospital and Medical School, Dundee, UK

[2]School of Nursing & Health Sciences, University of Dundee, Dundee, UK

[3]Institute of Health Research, University of Exeter Medical School, Exeter, UK

[4]Research, Development & Innovation, Royal Cornwall Hospitals NHS Trust, Truro, UK

[5]Re:Cognition Health, Plymouth, UK

[6]Institute for Applied Health Research, University of Birmingham, Birmingham, UK

[7]Cardiology Department, Sandwell & West Birmingham Hospitals NHS Trust, Birmingham, UK

For peer review only - http://bmjopen.bmj.com/site/about/guidelines.xhtml

[8]Department of Health Sciences, University of York, York, UK

[9]Research and Development, Anuerin Bevan University Health Board, St Woolos Hospital, Newport, UK

[10]Cardiology Department, Royal Cornwall Hospitals NHS Trust, Truro, UK

[11]Centre for Exercise and Rehabilitation Science, University Hospitals of Leicester NHS Trust, Glenfield Hospital, Leicester, UK

[12]REACH-HF Patient and Public Involvement Group, c/o Research, Development & Innovation, Royal Cornwall Hospitals NHS Trust, Truro, UK

[13]Sport and Health Sciences, University of Exeter, Exeter, UK

[14]Peninsula Clinical Trials Unit, University of Plymouth, Plymouth, UK

Word count: 3833; abstract – 290

For peer review only

For peer review only - http://bmjopen.bmj.com/site/about/guidelines.xhtml

Abstract

Introduction

Home-based cardiac rehabilitation may overcome suboptimal rates of participation. The overarching aim of this study was to assess the feasibility and acceptability of the novel REACH-HF rehabilitation intervention for patients with heart failure with preserved ejection fraction (HFpEF) and their caregivers.

Methods and results

Patients were randomised 1:1 to REACH-HF intervention plus usual care (intervention group) or usual care alone (control group). REACH-HF is a home-based comprehensive self-management rehabilitation programme that comprises patient and carer manuals with supplementary tools, delivered by trained healthcare facilitators over a 12-week period. Patient outcomes were collected by blinded assessors at baseline, 3 and 6 months post-randomisation and included health-related quality of life (primary) and psychological well-being, exercise capacity, physical activity and HF-related hospitalisation (secondary). Outcomes were also collected in caregivers.

We enrolled 50 symptomatic heart failure patients from Tayside, Scotland with a left ventricular ejection fraction ≥ 45% (mean age: 73.9 years, 54% female, 100% white British) and 21 caregivers. Study retention (90%) and intervention uptake (92%) were excellent. At 6 months, data from 45 patients showed a potential direction of effect in favour of the intervention group, including the primary outcome of Minnesota Living with Heart Failure Questionnaire total score (between group mean difference: -11.5, 95% confidence interval: -22.8 to 0.3). A total of 11 (4 intervention, 7 control) patients experienced a hospital admission over the 6 months follow up with 4 (control patients) of these admissions being HF-related. Improvements were seen in a number intervention caregiver mental health and burden compared to control.

Conclusions

Our findings support the feasibility and rationale for delivering the REACH-HF facilitated home-based rehabilitation intervention for patients with HFpEF and their caregivers and progression to a full multicentre randomised clinical trial to test its clinical and cost-effectiveness.

Key words: Heart Failure, Heart Failure with Preserved Ejection Fraction, Cardiac Rehabilitation, Randomised Controlled Trial, Complex Intervention, Caregivers.

Trial registration number: ISRCTN78539530

For peer review only - http://bmjopen.bmj.com/site/about/guidelines.xhtml

Strengths

- REACH-HF is the first comprehensive home-based, self-management cardiac rehabilitation intervention for HFpEF patients and their caregivers.
- The findings of this pilot study support the feasibility and acceptability of the home-based REACH-HF rehabilitation intervention in patients with HFpEF and their caregivers and indicate that it is feasible to recruit and retain participants in a randomised trial with follow-up.
- Potential favourable impacts of the REACH-HF intervention on caregiver mental health and measures of burden were observed in this pilot study.

Limitations

- This study was not designed or powered to definitively assess the efficacy or safety of the REACH-HF intervention in HFpEF.
- Generalisability of this study's findings is limited, given it was based in a single centre.

Introduction

Epidemiological data show that approximately half of those patients with clinical features of heart failure (HF) have preserved ejection fraction (HFpEF).[1] In contrast to HF with reduced ejection fraction (HFrEF), the prevalence of HFpEF is increasing.[2] Importantly, the substantial burden from HFpEF appears to be similar to HFrEF, measured by exercise intolerance, poor health-related quality of life (HRQoL), mortality, increased hospital admissions and higher healthcare costs.[3] Although drug and device therapy have helped to improve outcomes in HFrEF, prognosis in HFpEF remains unchanged, with no large-scale randomised trial demonstrating significant treatment benefits that alter the natural course of HFpEF or lower mortality.[4,5] However, systematic reviews and meta-analyses have shown promising evidence for the benefit of exercise-based cardiac rehabilitation (CR) in HFpEF.[6,7] A recent meta-analysis of 8 randomised trials in 317 HFpEF patients found exercise-based CR significantly improved exercise capacity and HRQoL compared to usual care.[7] The

For peer review only - http://bmjopen.bmj.com/site/about/guidelines.xhtml

CR programmes undertaken in these trials were predominantly group-based, supervised, and delivered in centre-based settings.

Participation of patients with HF in CR remains suboptimal.[8,9] A United Kingdom survey found that only 16% of CR centres provided a HF programme; commonly cited reasons for the lack of CR provision were a lack of resources and exclusion from commissioning agreements.[9] Two main reasons given by patients for failing to take part in CR are difficulties with regular attendance at their local hospital centre and reluctance to join group-based classes.[9]

There is increasing recognition of the possibility of alternative delivery models of CR, such as home-based programmes, in order to overcome suboptimal rates of CR uptake seen with HF.[10,11] Facilitated home-based CR has been shown to provide similar benefits to centre-based CR in terms of clinical and HRQoL outcomes at equivalent cost for those with HF and following myocardial infarction and revascularization.[11,12]

The Rehabilitation EnAblement in CHronic Heart Failure (REACH-HF) programme of research was designed to develop and evaluate a home-based comprehensive self-management rehabilitation intervention, including a self-care manual, an exercise programme, and facilitation by health professionals designed to improve self-management and HRQoL in people with HF. [13,14] In addition to REACH-HF intervention includes a 'Family and Friends Resource' designed to support caregivers.

The overarching aim of this study was to assess the feasibility of undertaking definitive randomised trial to assess the clinical effectiveness and cost-effectiveness of the REACH-HF intervention in patients with HFpEF and their caregivers. Specific objectives of the study were to: (1) assess the acceptability of the study design and procedures to participants (patients and caregivers); (2) assess feasibility and experience of the delivery of intervention for participants and healthcare professional facilitators; (3) identify barriers to participation in the intervention and study procedures; (4) inform a definitive study sample size; (5) assess methods for the collection of data including resource use and costs; and (6) assess the fidelity of the delivery of the REACH-HF intervention by healthcare professional facilitators.

For peer review only - http://bmjopen.bmj.com/site/about/guidelines.xhtml

Methods

The study design and methods have been described in the published study protocol.[14] The study is reported in accordance with the Consolidated Standards of Reporting Trials (CONSORT) extension for pilot trials.[15]

Design

The REACH-HFpEF pilot study was a single centre (Tayside, Scotland) two group randomised controlled trial with a parallel mixed methods feasibility evaluation and assessment of costs. Participants were individually randomised in a 1:1 ratio to the REACH-HF intervention plus usual care (intervention group) or usual care alone (control group). Given the nature of the REACH-HF intervention, it was not possible to blind participants or those involved in the provision of care. However, the statistician (FCW) undertaking the data analysis was blinded to treatment allocation and we also blinded researchers undertaking collection of outcome data to minimise potential bias. We assessed the fidelity of blinding by asking outcome assessors at each follow-up visit to guess patient group allocation. Unblinding of groups did not take place until after data analysis and the blinded results had been presented to the Trial Management Group and interpretation of results was agreed. Approvals were obtained from Scotland A Research Ethics Committee and the study was registered (ISRCTN 57596739).

Study population

The study population included patients and their caregivers. Participating patients were aged 18 years or older and had a confirmed diagnosis of HFpEF on echocardiography, radionuclide ventriculography or angiography (i.e. left ventricular ejection fraction ≥ 45% within the last 6 months prior to randomisation). Patients who had undertaken CR within 6 months prior to enrolment were excluded, as were patients with a contraindication to exercise testing or exercise training (with consideration of adapted European Society of Cardiology guidelines for HF).[14,16] Participating caregivers were aged 18 years or older and provided unpaid support to participating. Patients who did not have an identified caregiver were able to participate, as were those whose caregiver was not willing to participate in the study.

For peer review only - http://bmjopen.bmj.com/site/about/guidelines.xhtml

Intervention

The REACH-HF intervention is described in detail elsewhere[17] In summary, REACH-HF is a comprehensive self-management programme informed by evidence, theory, and service user perspective. It comprises the 'Heart Failure Manual' (REACH-HF Manual), Relaxation CD, chair-based exercise CD, a 'Progress Tracker' tool for patients, and a 'Family and Friends Resource' for caregivers. Participating patients and caregivers worked through the REACH-HF Manual over a 12-week period with facilitation by a two trained cardiac nurses. The facilitators provided support as needed of which at least one was to be face-to-face and two by telephone. The REACH-HF manual incorporates 5 core informative and interactive elements covering a wide range of topics relating to living with/adapting to living with HF, and includes:

1. a progressive exercise training programme, tailored according to initial fitness assessments, delivered as a walking programme or a chair-based exercise DVD, or a combination of the two (as selected by the patient);
2. managing stress/breathlessness/anxiety;
3. HF symptom monitoring;
4. taking medication; and
5. understanding HF (and why self-management helps).

The REACH-HF Manual was designed for patients with HFrEF (in terms of coverage of medication and explanations of condition). There was limited evidence to guide the development of the REACH-HF Manual for HFpEF patients. It was adapted for this pilot study to allow evaluation in patients with HFpEF. The majority of the self-management advice in all other sections of the REACH-HF Manual is relevant to all patients with HF and corresponds to national HF guidelines.[18.19] The core priorities for caregiver elements of the intervention were:

1. To facilitate improvement in patient HRQoL by helping them to achieve the core priorities for change.
2. To improve HRQoL for caregivers by acting to maintain their own health and well-being.

Usual care

Both intervention and control group patients received usual medical management for HF according to current guidelines.[18,19]

For peer review only - http://bmjopen.bmj.com/site/about/guidelines.xhtml

Outcome measures and follow up

We collected the following pilot study outcomes: recruitment rate for participants (patients and caregivers) across the various recruitment pathway; attrition and loss to follow up; completeness of participant outcome measures at follow up; fidelity of REACH-HF Manual delivery by intervention facilitators (sample of patient-facilitator contacts for sample of 6 patients were audio recorded and independently reviewed using a 13-item checklist (developed by CJG and JW) by two researchers (KS and Karen Coyle)); acceptability of the intervention (via face-to-face semi-structured interviews with purposive sample of 15 patients, 7 caregivers and both facilitators at the end of the intervention delivery period); and acceptability of study participation to participants (via interviews and questionnaire).

The following participant outcomes proposed for a future definitive trial were collected at baseline (pre-randomisation) and follow up at 4 and 6 months post randomisation:

Patients - disease-specific HRQoL (Minnesota Living with Heart Failure questionnaire (MLHFQ) (primary outcome);[20] and Heart Related Quality of Life (HeartQoL) questionnaire);[21] clinical events (all-cause mortality, hospital admission related to HF and not related to HF (relatedness was independently adjudicated by a panel of 3 cardiologists); exercise capacity (incremental shuttle walking test ISWT);[22] physical activity level (GeneActive accelerometry over a 7-day period);[23] psychological wellbeing (Hospital Anxiety and Depression Scale questionnaire, HADS);[24] generic HRQoL (EQ-5D-5L questionnaire);[25] Self-care of HF Index questionnaire (SCHFI);[26] healthcare utilisation (primary and secondary care contacts, social care contacts and relevant medication usage, reported by patient participants); and safety outcomes (serious adverse events).

Caregivers - Caregiver Burden Questionnaire-HF (CBQ-HF),[27] Caregiver Contribution to Self-care of HF Index questionnaire (CC-SCHFI);[26] Family Caregiver Quality of Life Scale questionnaire (FAMQOL);[28] Generic HRQoL (EQ-5D-5L);[25] and psychological wellbeing (HADS).[24]

Data analysis

Our planned recruitment target of 50 patients allowed us to achieve the feasibility aims and objectives of this study, i.e., an estimate of attrition, estimates of the

For peer review only - http://bmjopen.bmj.com/site/about/guidelines.xhtml

standard deviation (SD) of the primary and secondary outcomes to inform power for a future definitive trial, and sufficient numbers for qualitative interviews.

We report the mean and SD (or relevant summary statistics) for both groups for all patient and caregiver outcomes at each follow-up point and the mean (and 95% confidence interval (CIs)) for the between group difference in outcomes at 6-month follow-up adjusting for baseline outcome. Given the pilot nature of this trial, we do not report p-values for the comparison of outcomes between groups. All analyses are based on the intention-to-treat principle, i.e., according to the original randomisation and based on complete case data sets.

Data on patient resource use related to health and social care were collected using a standardised resource use questionnaire at baseline (for previous 6-months) and at 4 and 6 months follow-up. Unit costs per item of resource use were obtained from published estimates and where necessary inflated to 2016 prices using the Healthcare and Community Health Services index (see eTable 1).[29] These unit costs were then applied to the resource use reported at patient level to estimate the delivery costs associated with the REACH-HF manual, and the total costs associated with health and social care at baseline and over the 6-month follow-up. As with clinical outcome, costs are presented descriptively. EQ-5D-3L utilities were obtained using existing crosswalk values from EQ-5D-5L.[30] All outcomes and costs analyses were conducted using Stata (v14.2; College Station, TX, USA: StataCorp LP).

Patient, caregiver, and facilitator interviews were transcribed verbatim and analysed using thematic analysis.[31]

For peer review only

For peer review only - http://bmjopen.bmj.com/site/about/guidelines.xhtml

Results

Recruitment and retention of patients and caregivers and acceptability of trial design

Study enrolment, allocation, and follow-up of study participants are summarised in the CONSORT flow diagram shown in Figure 1. Between April 2015 and June 2016, 225 potential patients were approached and 50 were randomised (intervention group: 25; control group: 25) i.e. 22% (95% CI: 17% to 28%) of patients approached. The original forecast was a recruitment rate of 5 patients per month. However, the actual recruitment rate during the trial was 4.5 patients per month, resulting in a 1-month extension to the period of recruitment. A caregiver was recruited in connection with 21 (42%) patient participants (intervention group: 11; control group: 10).

At 6-month follow-up, 5 out 50 (10%, 95%: 3% to 22%) patients were lost to follow-up. Seventeen out of the 21 recruited caregivers provided follow up data at 6-months. Patients and caregivers rated a high level of satisfaction with their participation in the trial (see eTable 2).

Baseline characteristics of patients and caregivers

There was evidence of imbalance between intervention and control group patients in terms of their baseline demographic characteristics (see Table 1). Compared with the control group, the intervention group included a higher proportion of females, and lower proportions of patients with an ischaemic diagnosis, with atrial flutter/atrial fibrillation, and with chronic renal failure; also, the intervention group had a younger mean age. Caregivers were typically the partner or children of patients, were of a younger mean age than participating patients and predominantly female.

Table 1a Patient baseline demographic characteristics

Table 1b Caregiver baseline demographic characteristics

For peer review only - http://bmjopen.bmj.com/site/about/guidelines.xhtml

Table 1a. Patient baseline demographic characteristics

	**Intervention**<br>N = 25<br>Mean (SD) or N (percent)	**Control**<br>N = 25<br>Mean (SD) or N (percent)
Gender: male	9 (36)	14 (56)
Age (years)	71.8 (9.9)	76.0 (6.6)
BMI (kg^2/m)	32.1 (6.3)	32.2 (5.3)
Ethnic group: white	25 (100)	25 (100)
Relationship status:		
Single	4 (16)	2 (8)
Married	14 (56)	8 (32)
Divorced/civil partnership dissolved	1 (4)	3 (12)
Widowed	6 (24)	12 (48)
Domestic residence:		
Live alone	9 (36)	14 (56)
Spouse/partner only	14 (56)	8 (32)
Spouse/partner & child > 18 years	0 (0)	2 (8)
Other adult family members only	2 (8)	1 (4)
Smoking status:		
Never smoked	2 (8)	2 (8)
Ex-smoker	15 (60)	14 (56)
Current smoker	8 (32)	9 (36)
NYHA status:		
Class I	1 (4)	1 (4)
Class II	15 (60)	16 (64)
Class III	9 (36)	8 (32)
Class IV	0 (0)	0 (0)

For peer review only - http://bmjopen.bmj.com/site/about/guidelines.xhtml

For peer review only

Cause of heart failure:[1]		
Ischaemic	8 (32)	16 (64)
Non-ischaemic	16 (64)	8 (32)
Unknown	1 (4)	1 (4)
Number of comorbidities:		
0	7 (28)	12 (48)
1	15 (60)	6 (24)
2	3 (12)	4 (16)
3	0	2 (8)
4	0	1 (4)
Previous myocardial infarction	4 (16)	5 (20)
Previous atrial fibrillation/atrial flutter	6 (24)	13 (52)
Hypertension	18 (72)	14 (56)
Diabetes mellitus	9 (36)	6 (24)
Chronic renal impairment	3 (13)	10 (40)
Time since diagnosis of HF (years):		
< 1	6 (24)	4 (16)
1 to 2	7 (28)	6 (24)
> 2	12 (48)	15 (60)
Medication:		
Beta-blocker	18 (72)	13 (52)
Angiotensin 2 receptor antagonist	7 (28)	7 (28)
ACE inhibitor	11 (44)	14 (56)
Main activity:		
In employment or self-employment	0 (0)	1 (4)
Retired	22 (88)	24 (96)
Unemployed	2 (8)	0 (0)
Other	1 (4)	0 (0)

For peer review only - http://bmjopen.bmj.com/site/about/guidelines.xhtml

Education:		
Post school	7 (28)	7 (28)
Degree	5 (20)	5 (20
Pro-BNP levels:		
≤ 2000 pg/ml	23 (92)	22 (88)
> 2000 pg/ml	2 (8)	3 (12)

[1]Cause of HF determined by Principal Investigator

Table 1b. Caregiver baseline demographic characteristics

	**Intervention** N=11[1] Mean (SD) or N (percent)	**Control** N=10 Mean (SD) or N (percent)
Gender: male	3 (30)	2 (20)
Age (years)	59.3 (14.0)	64.8 (11.6)
Relationship to patient		
Partner	4 (40)	6 (60)
Son/daughter	3 (30)	4 (40)
Sibling	2 (20)	0 (0)
Friend	1 (10)	0 (0)

[1]One caregiver withdrew shortly after randomisation and did not provide baseline da

Completion of outcome measures by patients and caregivers and fidelity of blinding by outcome assessors

We collected data from 45/50 patients (90%, 95% CI: 78% to 97%) at 6-month follow-up on the MLHFQ, our proposed primary outcome. Levels of completion of patient secondary outcomes and caregiver outcomes were consistently high (≥ 76% of participants for all outcomes). The one exception was the ISWT, which had notably lower level of completion (35 (78%) patients at 4-month follow-up and 33 (73%) patients at 6-month follow-up).

Outcome assessors correctly guessed patient group allocation in 22% of cases (10/45) at 4 months and 20% of cases (19/45) at 6 months, indicating that blinding was likely to have been maintained.

For peer review only - http://bmjopen.bmj.com/site/about/guidelines.xhtml

For peer review only

Acceptability of patient, carers and facilitators of REACH-HF intervention and fidelity of intervention delivery by facilitators

Qualitative interviews and observations of the patient and caregivers interactions with the facilitator indicated high levels of satisfaction, acceptability and the feasibility of delivering the REACH HF Intervention in HFpEF patients (see eTable 3). One of the most highly valued elements of the REACH-HF by participants was the role of the facilitator, who was seen to act as an educator, a source of emotional support and reassurance as well as a motivator and enabler.

Of the 6 patients selected for inclusion, a total of ~45 hours of patient-facilitator interaction was used for analysis. Fidelity scoring indicated adequate delivery (defined as a score of 3 or more) for most aspects of the intervention by the two facilitators (see eTable 4). Of the 6 patients selected for inclusion, a total of ~45 hours of patient-facilitator interaction was used for analysis. Mean score for items 9 (addressing emotional consequences of being a caregiver) and 11 (caregiver health and well-being) was less than 3.

Patient adherence to REACH-HF intervention

Twenty three of the 25 (92%) intervention patients met our minimum adherence criteria of attendance i.e. attendance at the first face-to-face meeting with the facilitator and at least two further facilitator contacts (either face-to-face or telephone). In these patients, the mean number of facilitator contacts was 6.2 (SD: 1.6), the majority of which were face-to-face contacts (mean 5.1; SD: 1.5) and the remainder were telephone contacts (mean: 1.1, SD: 1.3) (see Table 4).

Table 4 REACH-HF intervention delivery: healthcare resource use and costs

For peer review only - http://bmjopen.bmj.com/site/about/guidelines.xhtml

Table 4. REACH-HF intervention delivery: healthcare resource use and costs

	**Number of patient contacts** Mean (SD)	**Duration of patient contacts contact (minutes)** Mean (SD)	**Duration facilitator non-contact planning (minutes)** Mean (SD)	**Duration facilitator travel (minutes)** Mean (SD)
Face to face contacts/patient	5.1 (1.5)	60.6 (29.6)	17.2 (24.4)	40.2 (37.4)
Telephone contacts/patient	1.1 (1.3)	7.7 (4.0)	8.0 (9.5)	
Total contacts/patients	6.2 (1.6)			
Total time, face to face contacts		308.9 (123.3)		
Total time, telephone contacts		8.8 (10.3)		
Total facilitator planning/non-contact time, face to face, minutes		87.4 (55.8)		
Total facilitator planning/non-contact time, telephone, minutes		9.1 (12.6)		
Overall total time input, time		414.2 (145.4)		
		**Cost per patient**[1] Mean (SD)		
Estimated total HF Facilitator cost,		£303.64 (£106.59)		

For peer review only

Other resource use/costs:<br>- consumables (1 x manual)<br>- DVDs (x 2, at £7.50 each)<br>- Distribution of HF Facilitator training costs, per participant[2]		 £25.00<br>£15.00<br>£18.97		
Estimated total delivery cost of HF-REACH intervention		£362.61		

[1]Unit costs – Staff: Staff grade equivalent to 'Community Nurse' (includes district nursing sister, district nurse) and Nurse Specialist (community), from Curtis and Burns, Unit Costs of Health and Social Care 2016, p141-142. Based on Agenda for Change band 6 (staff salary at £32,114 pa). Estimated cost per hour = £44 (Curtis and Burns, 2016); Includes salary, salary on-costs, overheads (management costs, and non-staff costs (including travel/transport]), capital overheads, and excludes costs for qualifications.

[2]Training cost per REACH HF Facilitator, specific to delivery of the REACH-HF intervention, are estimated at £1,897 (involving 3 days, i.e. 24 hours training at £44/hour; costs for Trainer/s per Trainee at £366, assuming 8 Trainees per 3 day course, and Trainers at Agenda for Change, Band 8a, £61/hr (Curtis and Burns, 2016); cost for REACH-HF Facilitator Manual at £400 each; plus estimate of consumables for training sessions). These costs are distributed across the first 100 participants/patients receiving the intervention, resulting in an estimate of £18.97 per participant.

Participant outcomes

Patients

Patient outcome results at baseline, and 3-month and 6-month follow-up, and between group differences at 6-month follow-up are shown in Table 2 (see eTable 5 for baseline-follow up within group changes). At 6 months, a number of patient outcomes potential direction of effect in favour of intervention, including MLWHF total score (between group mean difference: -11.5, 95% confidence interval (CI): -22.8 to 0.3), HeartQoL global score (0.5, 95% CI: 0.0 to 0.9), EQ-5D-3L utility index (0.11, 95% CI: -0.04 to 0.26), HADS depression score (-1.5, 95% CI: -3.4 to 0.3), and SCHFI maintenance score (9.5, 95% CI: 2.5 to 17.3). The direction of possible intervention effects were less clear for the outcomes of ISWT and level of physical activity.

For peer review only - http://bmjopen.bmj.com/site/about/guidelines.xhtml

At 6-months follow up, 11 (4 intervention, 7 control) patients experienced a hospital admission with 4 (all control) of these admissions being HF-related. All these serious adverse events were considered to be unrelated to the study processes or to the REACH-HF intervention. One control patient died related to HF shortly after the 6-month follow-up.

Table 2 Patient outcomes at baseline and follow-up

For peer review only

For peer review only - http://bmjopen.bmj.com/site/about/guidelines.xhtml

Table 2. Patient outcomes at baseline and follow-up

	**Baseline**		**4-month follow-up**		**6-month follow-up**		
	**Intervention** Mean (SD), N	**Control** Mean (SD), N	**Intervention** Mean (SD), N	**Control,** Mean (SD), N	**Intervention** Mean (SD), N	**Control** Mean (SD,) N	**Mean between group difference[1] (95% CI)**
Primary outcome							
MLHFQ, Overall	38.2 (27.6), 25	36.0 (26.5), 25	35.5 (28.3), 22	37.8 (27.9), 23	29.2 (25.8), 22	38.7 (30.1), 23	-11.5 (-22.8 to 0.3)
MLHFQ, Physical	21.6 (13.4), 25	19.8 (12.4), 25	19.4 (13.5), 22	20.7 (12.8), 23	16.2 (12.3), 21	20.3 (13.6), 23	-4.7 (-10.1 to 0.8)
MLHFQ, Emotional	7.8 (9.1), 25	7.8 (8.4), 25	8.0 (8.5), 22	9.1 (8.6), 23	6.8 (8.1), 21	9.0 (8.5), 23	-2.7 (-6.0 to 0.6)
Secondary outcomes							
HADS, Anxiety	5.6 (4.8), 25	6.1 (4.9), 25	5.7 (4.8), 22	6.4 (5.4), 23	5.5 (5.1), 21	6.0 (5.1), 23	-0.2 (-2.6 to 2.1)
HADS, Depression	6.2 (4.2), 25	5.6 (4.1), 25	5.6 (4.4), 22	6.6 (4.5), 23	5.4 (4.3), 21	6.9 (5.2), 23	-1.5 (-3.4 to 0.3)
Heart-QoL, Global	1.4 (0.8), 25	1.6 (0.9), 25	1.5 (1.0), 22	1.4 (1.0), 23	1.8 (0.8), 21	1.4 (1.1), 23	0.5 (0.0 to 0.9)

For peer review only - http://bmjopen.bmj.com/site/about/guidelines.xhtml

Heart-QoL, Physical	1.2 (0.8), 25	1.4 (1.0), 25	1.3 (1.0), 22	1.3 (1.0), 23	1.6 (0.8), 21	1.3 (1.1), 23	0.5 (0.0 to 1.0)
Heart-QoL, Emotional	2.0 (1.0), 25	2.0 (1.0), 25	2.0 (1.0), 22	1.9 (1.0), 23	2.2 (1.0), 21	1.8 (1.1), 23	0.3 (-0.1 to 0.8)
EQ-5D-3L, index score	0.57 (0.29), 25	0.58 (0.31), 24	0.60 (0.28), 22	0.52 (0.34), 23	0.65 (0.31), 21	0.55 (0.29), 23	0.11 (-0.04 to 0.26)
SCHFI, Maintenance	51.9 (13.9), 25	45.3 (16.5), 25	68.9 (14.9), 22	49.6 (14.4), 23	64.2 (12.8), 21	48.9 (14.3), 23	9.9 (2.5 to 17.3)
SCHFI, Management	37.6 (20.7), 23	37.8 (18.4), 18	48.9 (26.5), 19	32.6 (19.2), 17	45.0 (2.7), 14	37.6 (23.5) 15	8.0 (-8.9 to 25.0)
SCHFI, Confidence	60.4 (25.5), 25	56.9 (23.0), 25	65.2 (18.7), 22	49.5 (24.9), 23	62.1 (20.0), 21	53.4 (26.1), 23	6.6 (-6.7 to 19.9)
ISWT (metres)	183.6 (174.2), 25	157.6 (117.8), 23	218.9 (185.5), 18	178.2 (115.0), 17	224.7 (161.4), 17	183.8 (98.1), 16	-2.1 (-39.4 to 35.2)
Accelerometry, number of days/week with at least 10 minutes/day activity > 100mg	5.8 (2.3), 25	5.9 (2.0), 25	5.6 (2.4), 21	5.7 (1.9), 21	4.9 (2.7), 19	6.0 (2.1), 20	-0.4 (-1.3 to 0.5)

For peer review only - http://bmjopen.bmj.com/site/about/guidelines.xhtml

Accelerometry, sverage time/day at ≤ 20mg (mins)	1126 (98), 25	1090 (112), 25	1115 (110), 21	1103 (124), 21	1136 (101), 19	1098 (114), 20	-10 (-49 to 28)
Accelerometry, average time/day at 21 to 40mg (mins)	128 (33), 25	152 (39), 25	140 (38), 21	143 (36), 21	134 (37), 19	148 (41), 20	12 (-4 to 29)
Accelerometry, average time/day at 41 to 60mg (mins)	77 (27), 25	87 (29), 25	79 (29), 21	84 (33), 21	75 (25), 19	85 (27), 20	1 (-10 to 12)
Accelerometry, average time/day at 61 to 80mg (mins)	45 (20), 25	47 (20), 25	45 (23), 21	45 (21), 21	40 (20), 19	45 (19), 20	-1 (-9 to 6)
Accelerometry, average time/day at 81 to 100mg (mins)	25 (14), 25	25 (15), 25	25 (15), 21	25 (17), 21	22 (15), 19	25 (15), 20	-1 (-6 to 4)

For peer review only - http://bmjopen.bmj.com/site/about/guidelines.xhtml

Accelerometry, average time/day at > 100mg (mins)	39 (30), 25	40 (48), 25	36 (31), 21	39 (52), 21	32 (30), 19	39 (48), 20	-2 (-9 to 5)

[1]Mean between group differences (intervention minus control) adjusted for baseline values.

For peer review only

For peer review only - http://bmjopen.bmj.com/site/about/guidelines.xhtml

Caregivers

Caregiver outcome results at baseline and 3-month and 6-month follow-up are shown in Table 3 (see eTable 6 for within group results). There were indications of a favourable intervention effect for some outcomes including HADS depression and anxiety scores and CBQ-HF emotional and CC-SCHFI maintenance domain scores.

Table 3 Caregiver outcomes at baseline and follow-up

For peer review only

For peer review only - http://bmjopen.bmj.com/site/about/guidelines.xhtml

Table 3. Caregiver outcomes at baseline and follow-up

	Baseline		4-month follow-up		6-month follow-up		
	**Intervention** Mean (SD), N	**Control**, Mean (SD), N	**Intervention**, Mean (SD), N	**Control**, Mean (SD), N	**Intervention**, Mean (SD), N	**Control**, Mean (SD), N	**Mean between group difference[1] (95% CI)**
HADS, Anxiety	8.6 (5.4), 10	6.2 (5.5), 10	7.1 (7.0), 8	6.8 (3.0), 10	6.3 (6.2), 8	7.6 (4.7), 9	-3.4 (-6.6 to 0.2)
HADS, Depression	4.0 (4.0), 10	4.7 (4.3), 10	3.9 (3.2), 8	5.4 (3.8), 10	2.9 (3.4), 8	5.9 (3.4), 9	-2.3 (-5.1 to -0.5)
FAMQOL, Overall	61.4 (10.5), 10	56.9 (12.0), 10	60.0 (10.2), 8	54.3 (12.6), 10	56.8 (8.6), 8	54.0 (8.7), 9	-1.1 (-7.9 to 5.6)
FAMQOL, Physical	17.0 (2.6), 10	14.9 (3.3), 10	15.9 (2.9) 8	14.9 (3.1), 10	15.8 (1.8), 8	15.0 (2.2), 9	-1.2 (-2.7 to 0.3)
FAMQOL, Psychological	13.9 (5.3), 10	13.5 (5.2), 10	13.3 (4.5), 8	12.0 (4.2), 10	12.8 (5.0), 8	12.1 (3.6), 9	0.3 (-2.7 to 3.3)
FAMQOL, Social	16.6 (2.8), 10	15.8 (4.7), 10	16.3 (2.4), 8	14.8 (3.6), 10	15.6 (0.9), 8	14.8 (2.5), 9	0.0 (-1.6 to 1.5)
EQ-5D-3L, utility score	0.78 (0.19), 10	0.74 (0.28), 10	0.81(0.10), 8	0.75 (0.17), 10	0.77 (0.18), 8	0.67 (0.35), 9	0.07 (-0.08 to 0.22)
CBQ-HF, Physical	4.5 (5.9), 10	3.7 (4.7), 10	2.0 (4.1), 8	6.3 (6.0), 10	4.4 (7.3) 8	5.2 (5.8), 9	-1.5 (-4.1 to 1.1)

For peer review only - http://bmjopen.bmj.com/site/about/guidelines.xhtml

CBQ-HF, Emotional	17.4 (13.8), 10	18.8 (13.0), 10	15.1 (13.3), 8	20.3 (12.0), 10	15.4 (16.0, 8	22.3 (13.1), 9	-5.1 (-12.5 to 2.3)
CBQ-HF, Social Life	0.7 (1.2), 10	1.6 (2.0), 10	0.4 (0.7), 8	1.8 (2.1), 10	0.6 (1.1), 8	2.2 (2.5), 9	-0.8 (- 2.6 to 1.1)
CBQ-HF, Lifestyle	1.9 (2.3), 10	4.3 (3.2), 10	2.5 (3.1), 8	4.4 (3.3), 10	2.4 (3.2), 8	6.0 (4.5), 9	-1.4 (-4.7 to 1.9)
CC-SCHFI, Maintenance	22.0 (11.0), 10	30.3 (15.7), 10	34.2 (25.1), 8	31.7 (14.6), 10	36.3 (23.5), 8	40.7 (17.9), 9	1.5 (-19.1 to 22.2)
CC-SCHFI, Management	29.0 (21.6), 10	35.6 (14.7), 8	39.3 (28.2), 7	35.0 (18.5), 7	45.0 (13.2), 3	35.0 (19.1), 8	7.4 (-21.4 to 36.2)
CC-SCHFI, Confidence	33.9 (15.6), 10	29.6 (19.8), 10	35.4 (17.6), 8	20.0 (17.2), 10	38.2 (16.4), 8	33.3 (20.6), 9	2.6 (-16.0 to 21.2)

[1] Mean between group differences (intervention minus control) adjusted for baseline values.

For peer review only

For peer review only - http://bmjopen.bmj.com/site/about/guidelines.xhtml

Healthcare utilisation and intervention costs

The average cost of the REACH-HF intervention per patient was estimated to be £362.61. The intervention cost breakdown is provided in Table 4. The wider healthcare and societal utilisation and costs for intervention and control groups are summarised in eTable 7.

Discussion

The findings of this pilot study support the feasibility and acceptability of the home-based REACH-HF rehabilitation intervention in patients with HFpEF and their caregivers, and indicate that it is feasible to recruit and retain participants in a randomised trial of 6-months follow up. The intervention was well received by patients, caregivers, and healthcare facilitators and intervention adherence was good. At follow up, compared to control, a number of patient outcomes showed a potential direction of effect in favour of the intervention group, including our proposed primary outcome of disease-specific HRQoL - MLWHF. We also saw potentially favorable impacts of the REACH-HF intervention on caregiver mental health and measures of burden.

The promising results of this study support the emerging evidence of the impact of exercise-based CR interventions in HFpEF.[6,7] A recent meta-analysis of randomised trials (ranging in sample size from 25 to 198 patients) suggest improvements in exercise capacity and HRQoL following intervention compared with control.[7] However, these previous studies have predominantly been supervised and delivered in centre-based settings. Participation in centre-based CR has been sub-optimal, with national practice surveys indicating that fewer than 20% of eligible HF patients may be receiving exercise-based CR.[8] Therefore, there is increasing interest in home-based programmes that have the potential to overcome these suboptimal rates of CR participation seen with HF.[10,11] To our knowledge, REACH-HF is the first comprehensive self-management CR intervention for HFpEF patients and their caregivers that is home-based, with facilitation by healthcare professionals and whose development is informed by evidence, theory, and input from service users – patients and clinicians.

The mechanism by which CR improves HRQoL in HFpEF remains unclear.[32] Whilst exercise training has been shown to improve cardiac (systolic and diastolic) function in HFrEF patients, studies have failed to show such consistent benefits in HFpEF. [6,7]Instead exercise training may improve exercise tolerance in HFpEF through peripheral mechanisms leading to an improved oxygen extraction in the active skeletal muscles.[33] Such improvements are likely to improve patient physical capacity and hence the physical component of HRQoL. Poor mental health, including depression in

For peer review only - http://bmjopen.bmj.com/site/about/guidelines.xhtml

HF patients is common and may be under recognised and undertreated in cardiac populations such as HFpEF. This is supported by the baseline HADS scores in this study indicating mild to moderate symptoms of depression and anxiety in a proportion of patients (and caregivers). A recent Cochrane review has shown comprehensive CR, including elements of stress management and exercise training, can have significant positive effects in terms in reductions in depression and anxiety of myocardial infarction and post-revascularisation populations.[34] The observed trend towards a reduction depression and anxiety scores with the REACH-HF intervention, points toward a possible basis of improvement in the mental component of HRQoL.

This study has a number of limitations. Firstly, the study was not designed or powered to definitively assess the efficacy or safety of the REACH-HF intervention in HFpEF. Secondly, generalisability of this study's findings is limited, given it was based in a single centre. Thirdly, there was evidence of imbalances between and intervention and control groups in their demographic characteristics and outcome scores at baseline. Fourthly, patient and clinician blinding was not possible in this study because of the nature of the intervention, although we did demonstrate that it was possible to blind outcome assessors to group allocation. Given the pilot nature of this trial and these limitations, our findings should therefore be considered preliminary, and encouraging trends require confirmation in a larger, adequately powered clinical trial.

Implications for planning a future trial

Based on MLWHFQ total score as the primary outcome, a full trial comparing the REACH-HF plus usual versus usual care alone would require recruitment of 210 HFpEF patients per group. This estimate is based on detecting a minimum clinically important difference on the MLWHFQ of 5 points,[20] a standard deviation of 25 points (as seen in this pilot trial, see Table 1), a within patient correlation of 0.8 (between baseline and 6-month follow-up calculated from data from this pilot), and an assumed attrition rate of 10% (as seen in this pilot trial, see Figure 1), at 90% power and 5% alpha level.

Two issues raised in this pilot that deserve consideration for a full trial include the choice of exercise test and the assessment of patient adherence to the REACH-HF intervention. In interviews, a number of patients in this study expressed the opinion that they found undertaking the ISWT as an unpleasant experience; 12 of 45 (27%) patients were not able to undertake the ISWT at 6-month follow-up. This loss to follow up my have resulted in bias in our assessment of exercise capacity over time and in our comparison of groups. Assessing and ensuring adequate levels of intervention adherence is a challenge in self-directed home based interventions, such as REACH-

For peer review only - http://bmjopen.bmj.com/site/about/guidelines.xhtml

HF.[11] Levels of patient attendance at face-to-face or telephone contacts with healthcare facilitators indicated good levels of intervention adherence. Patients were also asked to document changes in their health behaviours in a 'Patient Tracker' diary over the duration of the study. We need to examine if these diaries support our conclusion of good intervention adherence seen from facilitator contacts. It will be important to revisit these two issues in the design and planning of a future full trial.

In summary, the findings from this pilot study indicate that the REACH-HF home-based comprehensive self-management CR intervention facilitated by healthcare professional is feasible, acceptable and suggests promising effects on HFpEF patient and caregiver outcomes. This pilot study will help inform the funding application for a fully powered multi-centre randomised trial to assess the clinical effectiveness and cost-effectiveness of the novel REACH-HF intervention in HFpEF patients and their caregivers.

For peer review only

Acknowledgements

We would like to thank the research nurses (Lynn Rutherford and Fatima Baig) intervention facilitators (Anona Cranston and Gillian Smith) and patients and their caregivers in Dundee and Tayside who participated in this study. We also wish to acknowledge: the data team at Peninsula Clinical Trials Unit (University of Plymouth, UK), Lisa Price (University of Exeter, UK) who assisted with the analysis of accelerometry data, Karen Coyle (University of Dundee) who assisted with qualitative interviews and intervention fidelity checking, and Louise Taylor and her team at the Heart Manual Office Heart Office (Astley Ainslie Hospital, Edinburgh) for their assistance with the preparation and development of the REACH-HF intervention.

Conflicts of interest

RST is the lead for the ongoing portfolio of Cochrane reviews of cardiac rehabilitation. RST and HMD are named Scientific Advisors for ongoing the National Institute of Health and Care Excellence (NICE) updated clinical guidelines for the management heart failure (CG108). HD is an ordinary member of the British Association for Cardiovascular Prevention and Rehabilitation (BACPR) council. All other co-authors declare no conflict of interest to declare.

Funding

This paper presents independent research funded by the National Institute for Health Research (NIHR) under its Programme Grants for Applied Research Programme (Grant Reference Number RP-PG-1210-12004). NB, CA, CJG and RST are also supported by the National Institute for Health Research (NIHR) Collaboration for Leadership in Applied Health Research and Care (CLAHRC) South West Peninsula at the Royal Devon and Exeter NHS Foundation Trust; KJ by CLAHRC West Midlands and SS by CLAHRC East-Midlands. The views expressed are those of the authors and not necessarily those of the NHS, the NIHR or the Department of Health.

For peer review only - http://bmjopen.bmj.com/site/about/guidelines.xhtml

PERSPECTIVES

COMPETENCY IN MEDICAL KNOWLEDGE

The present findings support that patients with HFpEF have a substantial burden with exercise intolerance and a poor health related quality of life

For peer review only

For peer review only - http://bmjopen.bmj.com/site/about/guidelines.xhtml

References

1. Dunlay SM, Roger VL, Redfield MM. Epidemiology of heart failure with preserved ejection fraction. *Nat Rev Cardiol.* 2017 May 11.
2. Lam CSP, Donal E, Kraigher-Krainer E, et al Epidemiology and clinical course of heart failure with preserved ejection fraction. *Eur J Heart Fail.* 2011;13:18-28.
3. Liao L, Anstrom KJ, Gottdiener JS, et al Long-term costs and resource use in elderly participants with congestive heart failure in the Cardiovascular Health Study. *Am Heart J.* 2007;153:245-52.
4. Holland DJ, Kumbhani DJ, Ahmed SH, et al Effects of treatment on exercise tolerance, cardiac function, and mortality in heart failure with preserved ejection fraction. A meta-analysis. *J Am Coll Cardiol.* 2011;57:1676-86.
5. Komajda M, Isnard R, Cohen-Solal A, et al. Effect of ivabradine in patients with heart failure with preserved ejection fraction: the EDIFY randomized placebo-controlled trial. *Eur J Heart Fail.* 2017 Apr 30. doi: 10.1002/ejhf.876. [Epub ahead of print]
6. Taylor RS, Davies EJ, Dalal HM, et al Effects of exercise training for heart failure with preserved ejection fraction: a systematic review and meta-analysis of comparative studies. *Int J Cardiol.* 2012;162:6-13.
7. Chan E, Giallauria F, Vigorito C, et al Exercise training in heart failure patients with preserved ejection fraction: a systematic review and meta-analysis. *Monaldi Arch Chest Dis.* 2016;86:759.
8. Dalal HM, Wingham J, Palmer J, et al Why do so few patients with heart failure participate in cardiac rehabilitation? A cross-sectional survey from England, Wales and Northern Ireland. *BMJ Open.* 2012;2:e000787.
9. Beswick AD, Rees K, Griebsch I, et al Provision, uptake and cost of cardiac rehabilitation programmes: improving services to under-represented groups. *Health Technol Assess.* 2004;8(41):iii-iv, ix-x, 1-152.
10. Dalal H, Taylor R. Telehealth technologies could improve suboptimal rates of participation in cardiac rehabilitation. *Heart.* 2016;102:1155-6.
11. Dalal HM, Zawada A, Jolly K, et al Home based versus centre based cardiac rehabilitation: Cochrane systematic review and meta-analysis. *BMJ.* 2010;340.
12. Buckingham SA, Taylor RD, Jolly K, et al Home-based versus centre-based cardiac rehabilitation: abridged Cochrane systematic review and meta-analysis. *Open Heart.* 2016;3(2).
13. Taylor RS, Hayward C, Eyre V, et al Clinical effectiveness and cost-effectiveness of the Rehabilitation Enablement in Chronic Heart Failure (REACH-HF) facilitated self-care rehabilitation intervention in heart failure

For peer review only

For peer review only - http://bmjopen.bmj.com/site/about/guidelines.xhtml

patients and caregivers: rationale and protocol for a multicentre randomised controlled trial. *BMJ Open.* 2015;5(12):e009994.

14. Eyre V, Lang CC, Smith K, et al Rehabilitation Enablement in Chronic Heart Failure—a facilitated self-care rehabilitation intervention in patients with heart failure with preserved ejection fraction (REACH-HFpEF) and their caregivers: rationale and protocol for a single-centre pilot randomised controlled trial. *BMJ Open*. 2016;6(10).
15. Eldridge SM, Chan CL, Campbell MJ, et al CONSORT 2010 statement: extension to randomised pilot and feasibility trials. *BMJ.* 2016;355.
16. Piepoli MF, Conraads V, Corra U, et al Exercise training in heart failure: from theory to practice. A consensus document of the Heart Failure Association and the European Association for Cardiovascular Prevention and Rehabilitation. *Eur J Heart Fail.* 2011;13:347-57.
17. Greaves CJ, Wingham J, Deighan C, et al Optimising self-care support for people with heart failure and their caregivers: development of the Rehabilitation Enablement in Chronic Heart Failure (REACH-HF) intervention using intervention mapping. *Pilot Feasibility Studies*. 2016;2:37.
18. McMurray JJ, Adamopoulos S, Anker SD, et al ESC guidelines for the diagnosis and treatment of acute and chronic heart failure 2012: the task force for the diagnosis and treatment of acute and chronic heart failure 2012 of the European Society of Cardiology. Developed in collaboration with the Heart Failure Association (HFA) of the ESC. *Eur Heart J.* 2012;33.
19. NICE. *Chronic heart failure: Management of chronic heart failure in adults in primary and secondary care*. [Available from: https://www.nice.org.uk/guidance/cg108]
20. Rector T, Kubo S, Cohn J. Patients' self-assessment of their congestive heart failure. Part 2: Content, reliability and validity of a new measure, the Minnesota Living with Heart Failure questionnaire. *Heart Failure.* 1987;Oct/Nov:198-209.
21. Oldridge N, Hofer S, McGee H, et al The HeartQoL: part II. Validation of a new core health-related quality of life questionnaire for patients with ischemic heart disease. *Eur J Prev Cardiol.* 2014;21:98-106.
22. Pulz C, Diniz RV, Alves AN, et al Incremental shuttle and six-minute walking tests in the assessment of functional capacity in chronic heart failure. *Can J Cardiol.* 2008;24:131-5.
23. van den Berg-Emons HJ, Bussmann JB, Balk AH, et al Validity of ambulatory accelerometry to quantify physical activity in heart failure. *Scand J Rehabil Med.* 2000;32:187-92.

For peer review only - http://bmjopen.bmj.com/site/about/guidelines.xhtml

24. Zigmond AS, Snaith RP. The hospital anxiety and depression scale. *Acta Psychiatr Scand.* 1983;67:361-70.
25. Devlin N, Shah K, Feng Y, et al *Valuing Health-Related Quality of Life: An EQ-5D-5L Value Set for England.* Office for Health Economics; 2016. Contract No.: 16/01.
26. Riegel B, Lee CS, Dickson VV, et al An Update on the Self-Care of Heart Failure Index. *J Cardiovasc Nurs.* 2009;24:485-97.
27. Humphrey L, Kulich K, Deschaseaux C, et al The Caregiver Burden Questionnaire for Heart Failure (CBQ-HF): face and content validity. *Health Qual Life Outcomes.* 2013;11:84.
28. Nauser JA, Bakas T, Welch JL. A New instrument to measure quality of life of heart failure family caregivers. *J Cardiovasc Nurs.* 2011;26
29. Curtis L, Burns A. *Unit Costs of Health & Social Care. Personal Services Research Unit.* [Available from: http://www.pssru.ac.uk/project-pages/unit-costs/2016/]
30. van Hout B, Janssen MF, Feng YS, et al Interim scoring for the EQ-5D-5L: mapping the EQ-5D-5L to EQ-5D-3L value sets. *Value in Health.* 2012;15:708-15.
31. Leavy P. *The Oxford handbook of qualitative research*: Oxford University Press; 2014.
32. Nolte K, Herrmann-Lingen C, Wachter R, et al Effects of exercise training on different quality of life dimensions in heart failure with preserved ejection fraction: the Ex-DHF-P trial. *Eur J Prev Cardiol.* 2015;22:582-93. 13.
33. Kitzman DW, Brubaker PH, Herrington DM, et al Effect of endurance exercise training on endothelial function and arterial stiffness in older patients with heart failure and preserved ejection fraction: a randomized, controlled, single-blind trial. *J Am Coll Cardiol*. 2013;62:584–592.
34. Richards SH, Anderson L, Jenkinson CE, et al Psychological interventions for coronary heart disease. *Cochrane Database Syst Rev.* 2017;4:CD002902

For peer review only

Data Sharing

The authors confirm that all data underlying the findings are fully available without restriction. The authors have made the clinical and economic data set available through the University of Exeter's Institutional Repository – Open Research Exeter (see https://ore.exeter.ac.uk). Access to these data is permitted but controlled through requests made via the repository to the chief investigator (Professor Taylor: r.s.taylor @exeter.ac.uk). Although use is permitted, this will be on the basis that the source of

For peer review only - http://bmjopen.bmj.com/site/about/guidelines.xhtml

the data is acknowledged (including the funder) and it includes reference to the data set 'handle'.

For peer review only

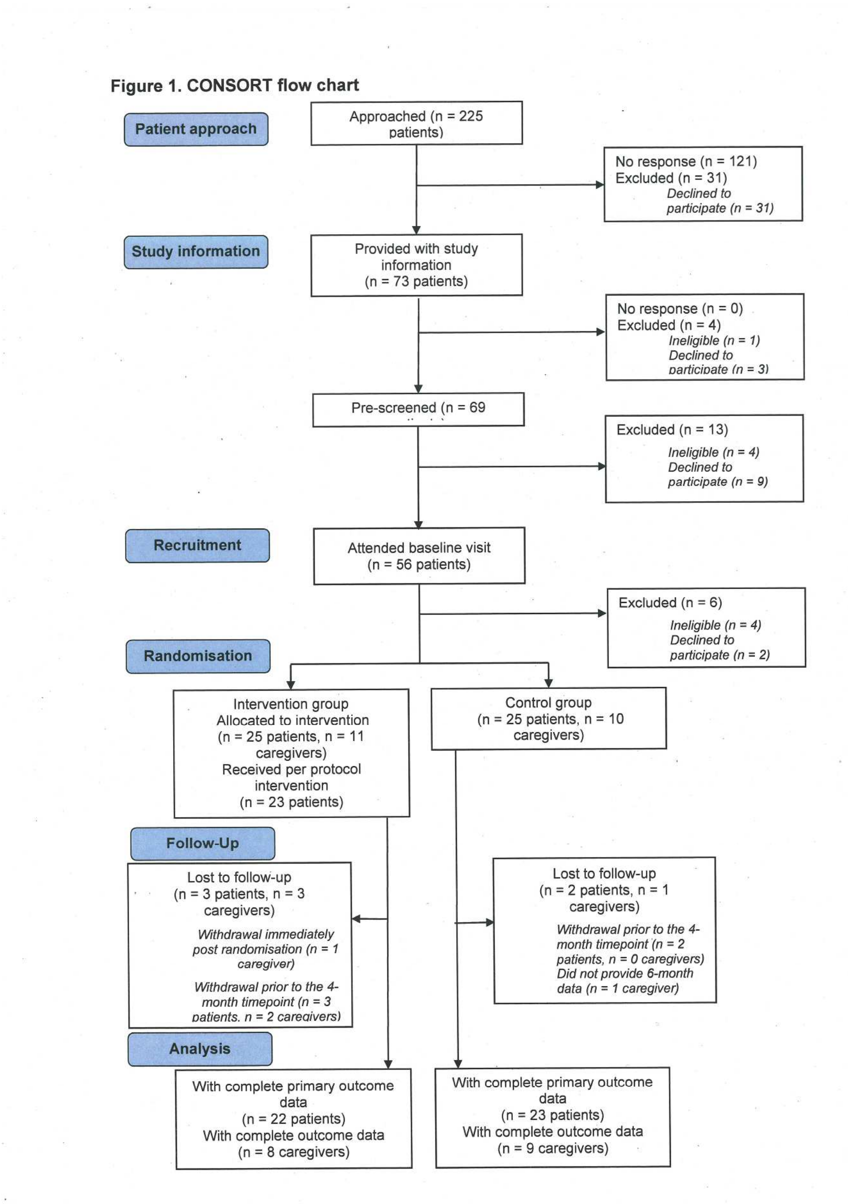

Figure 1. CONSORT flow chart

296x419mm (300 x 300 DPI)

eTable1. Unit costs

Resource use/Item	Unit cost 2016 £	Source
**Primary Care cost per visit/appointment**		
GP (surgery)	£31.00	Curtis and Burns, 2016
GP (home)	£74.98	Curtis and Burns, 2015
GP (phone)	£22.29	Curtis and Burns, 2015
Practice nurse (surgery)	£11.11	Curtis and Burns, 2016, Curtis and Burns, 2015
Practice nurse (home)	£18.80	Curtis and Burns, 2016, Curtis and Burns, 2015, Curtis, 2010.
Practice nurse (phone)	£4.30	Curtis and Burns, 2016, Curtis and Burns, 2015
Heart failure nurse	£22.11	Curtis and Burns, 2016,
Physiotherapist	£77.52	Curtis and Burns, 2016, Curtis, 2010.
Occupational therapist	£71.40	Curtis and Burns, 2016, Curtis, 2010.
Community/district nurse	£39.51	Curtis and Burns, 2016, Curtis, 2010.
Health visitor	£27.22	Curtis and Burns, 2015, Curtis, 2010.
Other primary/community service	£22.11	Curtis and Burns, 2016,
**Secondary care cost per event**		
Hospital admission (HF)	£4,668.66	Department of Health, 2016
Hospital admission (non-HF)	£3,966.57	Zannad *et al.* 2011, Department of Health, 2016
Hospital admission (overall)	£4,282.51	Combination of HF and non-HF admission cost, weighted according to admissions recorded in pilot
A&E attendance	£137.82	Department of Health, 2016
Day hospital attendance	£319.33	Department of Health, 2016
Outpatient cardiology appointment	£135.68	Department of Health, 2016
Outpatient cardiac or HF nurse	£102.96	Department of Health, 2016
Other outpatient appointment	£116.54	Department of Health, 2016

For peer review only - http://bmjopen.bmj.com/site/about/guidelines.xhtml

**Social & community care visits:**		
Social worker	£79.00	Curtis and Burns, 2016
Home care /home help	£12.00	Curtis and Burns, 2016
Voluntary agency	£10.00	Curtis and Burns, 2016
Day care	£46.00	Curtis and Burns, 2016
Drop in club	£13.00	Curtis and Burns, 2016
**Medications** (estimated 6-month cost per person)		
Angiotension 2-receptor antagonist	£15.09	Joint Formulary Committee 2017, OpenPrescribing.net
ACE inhibitor	£6.92	Joint Formulary Committee 2017, OpenPrescribing.net
Aldosterone receptor antagonist	£63.05	Joint Formulary Committee 2017, OpenPrescribing.net
Anti-coagulant	£8.34	Joint Formulary Committee 2017, OpenPrescribing.net
Beta-blocker	£6.15	Joint Formulary Committee 2017, OpenPrescribing.net
Digoxin	£18.00	Joint Formulary Committee 2017, OpenPrescribing.net
Ivabradine	£258.24	Joint Formulary Committee 2017, OpenPrescribing.net
Loop diuretic	£7.96	Joint Formulary Committee 2017, OpenPrescribing.net
Nitrate + hydralazine	£589.60	Joint Formulary Committee 2017, OpenPrescribing.net
Thiazide diuretic	£9.61	Joint Formulary Committee 2017, OpenPrescribing.net
**Patient & Caregiver Time** cost per unit		
Caregiver time, hour	£24.00	Curtis and Burns, 2016
Non-caregiver time, hour	£24.00	Curtis and Burns, 2016
Caregiver time off work, per day	£122.31	HM Revenue & Customs, 2017
Non-caregiver time off work, per day	£122.31	
Patient time off work, per day	£96.15	HM Revenue & Customs, 2017

For peer review only - http://bmjopen.bmj.com/site/about/guidelines.xhtml

References

Curtis L, Burns A. *Unit Costs of Health & Social Care 2016*. Personal Social Services Research Unit, The University of Kent. 2016.

Curtis L, Burns A. *Unit Costs of Health & Social Care 2015*. Personal Social Services Research Unit, The University of Kent. 2015.

Curtis L. *Unit Costs of Health & Social Care 2010*. Personal Social Services Research Unit, The University of Kent. 2010.

Department of Health, *NHS reference costs* 2015 to 2016, 2016.

HM Revenue & Customs, *Distribution of median and mean income and tax by age range and gender*: 2014 to 2015, UK National Statistics. 2017

BNF. Joint Formulary Committee. *British National Formulary* (online) London: BMJ Group and Pharmaceutical Press <http://www.medicinescomplete.com> [Accessed on 22.06.2017]

For peer review only - http://bmjopen.bmj.com/site/about/guidelines.xhtml

eTable 2. Patient and caregiver acceptability with trial participation at 6-months follow up

What was your overall impression of taking part in the study?	Very good or good N (%)	Acceptable N (%)	Poor or very Poor N (%)
**Patients**			
Intervention group, N = 21	19 (90)	2 (10)	0 (0)
Control group, N = 23	23 (100)	0 (0)	0 (0)
Both groups, N =44	42 (95)	2 (5)	0 (0)
**Caregivers**			
Intervention group, N = 8	7 (88)	1 (12)	0 (0)
Control group, N = 9	5 (55)	4 (45)	0 (0)
Both groups, N = 17	13 (72)	5 (28)	0 (0)

For peer review only

For peer review only - http://bmjopen.bmj.com/site/about/guidelines.xhtml

eTable 3. Patients and caregivers acceptability of REACH-HF intervention

The following are verbatim quotes of the positive experiences of the REACH-HF from patients and their caregivers.

Patient: *"I felt like giving her [the REACH-HF intervention facilitator] a hug to say thanks…you don't know what you've done for me… reach doesn't know what they have done for me" …. "Yeh so if anyone is listening to this and I hope 'youse' are and you are wanting to go on this programme, please go on it!!"*

Exercise

Caregiver: *"Yes, it was very helpful [exercise programme]. It really was. Helpful for me, as I say cos I started going out walking. We did the exercises…I'd never seen (my husband) and I laugh so much doing the exercises. You know, we had great fun. And the lady's straight face and…he would…he would put on funny poses and we laughed and we laughed. We thought…you know, we haven't laughed like that for a long, long time, you know. And it was really good. It really was."*

Patient: *After I think it was 9 weeks every single day I was trying my damdest to get past this, but I could not get past the (chair based exercise) warm up thing, so I said the facilitator I'm going to have to stop this (exercise) …. And she went 'no if you can't do that what do you love doing?' I says I love walking so she said 'right if you want to go out for a walk lets go out for a walk'…'*

Role of facilitator (education, support and reassurance)

Patient: *"I think that…reading the manual, talking to 'the nurse ', was very helpful for me in so many different ways. Helping me to understand heart failure….she encouraged me to go out walking…. Just the reassurance that things were better, that there was somebody there that was willing to, erm, say, well, okay, you're doing well. Even just the smallest amount of encouragement. And 'my husband' always felt better after the facilitator went away. Because she felt…almost like a little security blanket, if you want to say. That somebody was there, somebody was asking."*

Facilitator as motivator

For peer review only - http://bmjopen.bmj.com/site/about/guidelines.xhtml

Patient: *'she was wonderful, encouraged me to do more walking and so on and I knew I could do it '*

Supporting behavioural change

Patient: *I tried to watch what I am eating more, my diet I take far more care …. I'm eating a lot more fish and vegetables rather than meat .*

Emotional support for patents and caregivers

Patient: "I'd pulled myself in I was really very inward and they were all saying you should go out with your friends ,or do this, or have them up…..I think being able to speak about it was helpful because that's not me."

Caregiver: *"What I've found about this Programme was….the nurse that came. You could talk it through. After talking to her, I didn't have quite so bad a fear of it [heart failure]. You could tell her how frightened you were,..it's nice to have someone professional to say, well, look, okay, that's that day. I didn't actually realise that until she came, how good it was to actually sit and openly speak about it and openly say, well, ask advice and things. It was lovely having her. You know, it was just a support."*

Caregiver: "I think maybe it's helped him think I can live with this … you know it's not – it doesn't mean the end of things"

For peer review only - http://bmjopen.bmj.com/site/about/guidelines.xhtml

eTable 4. Fidelity of intervention delivery

	Item 1 Involve-ment	Item 2 Assess-ment	Item 3 Plan	Item 4 Under-stand	Item 5a Support – why to change	Item 5b Review	Item 6 Physical activity	Item 7 Emotion	Item 8 Medic-ation	Item 9 Care-giver	Item 10 Care-giver emotion	Item 11 Care-giver well-being	Item 12 Closure
**N patients**	6	6	6	6	6	6	6	6	6	6	6	6	6
**Mean score**	3.3	4.0	3.7	4.5	3.2	3.4	4.5	5.5	5.0	2.5	4.5	2.1	4.0

For peer review only - http://bmjopen.bmj.com/site/about/guidelines.xhtml

eTable 5. Within group difference in patient outcomes between baseline, and 4- and 6-month follow-up

	4-month follow-up vs. baseline Within group mean difference (95% CI)		6-month follow-up vs. baseline Within group mean difference (95% CI)	
	**Intervention**	**Control**	**Intervention**	**Control**
Primary outcome				
MLHFQ, Overall	-2.0 (-9.2 to 5.2)	3.0 (-4.7 to 10.7)	-8.3 (-16.8 to 0.1)	3.9 (-4.9 to 12.6)
MLHFQ, Physical	-1.1 (-4.5 to 2.3)	2.0 (-1.8 to 5.9)	-3.3 (-7.3 to 0.7)	1.6 (-2.7 to 5.9)
MLHFQ, Emotional	-0.6 (-2.7 to 1.5)	1.5 (-1.1 to 4.1)	-1.6 (-3.9to 0.7)	1.3 (-1.5 to 4.2)
Secondary outcomes				
HADS, Anxiety	0.0 (-1.4 to 1.3)	0.7 (-0.8 to 2.1)	0.1 (-1.5 to 1.8)	0.3 (-1.6 to 2.2)
HADS, Depression	-0.4 (-1.7 to 0.8)	1.0 (0.1 to 2.0)	-0.2 (-1.2 to 0.7)	1.3 (-0.2 to 2.8)
Heart-QoL, Global	0.0 (-0.2 to 0.3)	-0.2 (-0.5 to 0.0)	0.3 (0.0 to 0.5)	-0.2 (-0.6 to 0.1)
Heart-QoL, Physical	0.1 (-0.2 to 0.3)	-0.2 (-0.5 to 0.0)	0.3 (0.0 to 0.6)	-0.2 (-0.6 to 0.1)
Heart-QoL, Emotional	0.0 (-0.3 to 0.2)	-0.2 (-0.5 to 0.2)	0.1 (-0.1 to 0.4)	-0.2 (-0.6 to 0.2)
EQ-5D-3L, utility score	0.01 (-0.1 to 0.12)	-0.06 (-0.12 to -0.01)	0.05 (-0.08 to 0.18)	-0.03 (-0.12 to 0.07)
SCHFI, Maintenance	15.5 (9.4 to 21.5)	5.8 (1.1 to 10.6)	9.8 (4.5 to 14.8)	5.1 (-1.5 to 11.8)
SCHFI, Management	12.1 (1.3 to 22.9)	-5.4 (-14.9 to 4.2)	8.6 (-4.4 to 21.6)	-1.0 (-14.5 to 12.5)
SCHFI, Confidence	3.5 (-10.0 to 17.0)	-7.0 (-15.4 to 1.4)	0.2 (-10.4 to 10.8)	-3.1 (-15.3 to 9.0)
ISWT (metres)	5.0 (-27.9 to 37.9)	-12.9 (-41.3 to 15.4)	-7.9 (-44.6 to 28.7)	4.1 (-17.3 to 25.5)
Accelerometry, average time/day at ≤ 20mg	-9 (-36 to 18)	26 (5 to 48)	8 (-14 to 30)	26 (-5 to 60)

For peer review only - http://bmjopen.bmj.com/site/about/guidelines.xhtml

For peer review only

Accelerometry, average time/day at 21 to 40mg	11 (1 to 20)	-13 (-22 to -3)	5 (-3 to 13)	-11 (-25 to 2)
Accelerometry, average time/day at 41 to 60mg	2 (-6 to 10)	-6 (-12 to 0)	-2 (-8 to 5)	-7 (-17 to 3)
Accelerometry, average time/day at 61 to 80mg	0 (-5 to 6)	-3 (-7 to 1)	-4 (-10 to 2)	-4 (-10 to 2)
Accelerometry, average time/day at 81 to 100mg	0 (-3 to 3)	-1 (-4 to 1)	-2 (-5 to 1)	-1 (-5 to 2)
Accelerometry, average time/day at > 100mg	-4 (-9 to 1)	-3 (-6 to 0)	-5 (-11 to 1)	-4 (-8 to 1)

For peer review only

For peer review only - http://bmjopen.bmj.com/site/about/guidelines.xhtml

eTable 6. Within group difference in caregiver outcomes between baseline, and 4- and 6-month follow-up

	4-month follow-up vs. baseline Within group mean difference (95% CI)		6-month follow-up vs. baseline Within group mean difference (95% CI)	
	**Intervention**	**Control**	**Intervention**	**Control**
HADS, Anxiety	-2.1 (-5.4 to 1.1)	0.6 (-2.2 to 3.4)	-3.0 (-5.5 to -0.5)	0.9 (-1.5 to 3.3)
HADS, Depression	0.3 (-2.2 to 2.7)	0.7 (-1.6 to 3.0)	-0.8 (-2.6 to 1.1)	0.9 (-2.2 to 4.0)
FAMQOL, Overall	-3.0 (-10.4 to 4.4)	-2.6 (-6.7 to 1.5)	-6.3 (-13.1 to 0.6)	-1.9 (-7.8 to 3.9)
FAMQOL, Physical	-1.8 (-4.1 to 0.6)	0.0 (-2.3 to 2.3)	-1.9 (-3.3 to -0.4)	0.6 (-0.6 to 1.7)
FAMQOL, Psychological	-0.6 (-3.0 to 1.8)	-1.5 (-3.8 to 0.8)	-1.1 (-3.7 to 1.5)	-1.2 (-4.0 to 1.6)
FAMQOL, Social	-1.4 (-3.7 to 1.0)	-1.0 (-3.5 to 1.5)	-2.0 (-3.4 to -0.6)	-0.6 (-3.1 to 2.0)
EQ5D-3L, utility score	0.01 (-0.05 to 0.07)	0.01 (-0.10 to 0.12)	-0.03 (-0.12 to 0.07)	-0.08 (-0.19 to 0.02)
CBQ-HF, Physical	-2.4 (-5.6 to 0.8)	2.6 (-0.7 to 5.9)	0.0 (-2.4 to 2.4)	1.4 (-0.1 to 3.0)
CBQ-HF, Emotional	-1.1 (-5.0 to 2.8)	1.5 (-3.1 to 6.1)	-0.9 (-5.1 to 3.4)	4.0 (-2.4 to 10.4)
CBQ-HF, Social Life	-0.1 (-0.4 to 0.2)	0.2 (-0.8 to 1.2)	0.1 (-0.4 to 0.7)	0.6 (-1.1 to 2.2)
CBQ-HF, Lifestyle	0.8 (-1.2 to 2.7)	0.1 (-1.5 to 1.7)	0.6 (-1.4 to 2.7)	2.0 (-0.4 to 4.4)
CC-SCHFI, Maintenance	13.8 (-6.0 to 33.5)	1.4 (-5.6 to 8.4)	15.9 (-2.9 to 34.6)	11.9 (0.6 to 23.1)
CC-SCHFI, Management	5.0 (-10.1 to 20.1)	7.5 (-11.5 to 26.5)	5.0 (-27.9 to 37.9)	-1.4 (-19.3 to 16.4)
CC-SCHFI, Confidence	2.1 (-13.4 to 17.6)	-9.6 (-23.7 to 4.6)	4.9 (-11.1 to 20.8)	5.4 (-10.9 to 21.8)

For peer review only - http://bmjopen.bmj.com/site/about/guidelines.xhtml

For peer review only

For peer review only - http://bmjopen.bmj.com/site/about/guidelines.xhtml

Table e7. Wider healthcare and societal utilisation at 6-months follow up

	Intervention		Control	
	Appointments/ visits per person	Cost £ per person	Appointments/ visits per person	Cost £ per person
	mean (SD) N	mean (SD)	mean (SD) N	mean (SD)
**Primary Care Appointments**				
GP (surgery)	5.36 (7.68) 22	£166.16 (£238.08)	2.78 (2.04) 23	£86.18 (£63.24)
GP (home)	0.45 (0.91) 22	£33.74 (£68.24)	0.61 (2.29) 23	£45.74 (£171.71)
GP (phone)	0.64 (1.29) 22	£14.27 (£28.76)	0.91 (3.36) 23	£20.29 (£74.90)
Practice nurse (surgery)	2.77 (2.69) 22	£30.77 (£29.88)	2.61 (2.52) 23	£28.99 (£27.99)
Practice nurse (home)	0.09 (0.43) 22	£1.69 (£8.08)	0.00 (0.00) 23	£0.00 (£0.00)
Practice nurse (phone)	0.27 (0.94) 22	£1.16 (£4.04)	0.39 (1.88) 23	£1.68 (£8.08)
Heart failure nurse	0.00 (0.00) 22	£0.00 (£0.00)	0.00 (0.00) 23	£0.00 (£0.00)
Physiotherapist	2.73 (12.79) 22	£211.62 (£984.45)	1.00 (3.80) 23	£77.52 (£294.56)
Occupational therapist	0.00 (0.00) 22	£0.00 (£0.00)	0.52 (2.50) 23	£39.05 (£187.74)
Community/district nurse	0.05 (0.21) 22	£1.98 (£8.30)	0.39 (1.88) 23	£15.41 (£74.27)
Health visitor	0.00 (0.00) 22	£0.00 (£0.00)	0.00 (0.00) 23	£0.00 (£0.00)
Primary Care Total	12.36 (17.84) 22	£461	9.22 (11.10) 23	£315
**Secondary care**				
Hospital admission	0.18 (0.50) 22	£770.85 (£2141.25)	0.30 (0.63) 23	£1284.75 (£2697.98)
A&E attendance	0.00 (0.00) 22	£0.00 (£0.00)	0.09 (0.29) 23	£12.40 (£39.97)

For peer review only - http://bmjopen.bmj.com/site/about/guidelines.xhtml

Day hospital attendance	0.32 (0.72) 22	£102.18 (£229.92)	0.04 (0.21) 23	£12.77 (£67.06)
Outpatient cardiology appointment	0.41 (0.67) 2	£55.63 (£90.90)	0.57 (1.08) 23	£77.34 (£146.53)
Outpatient cardiac or HF nurse	0.05 (0.21) 22	£5.15 (£21.62)	0.00 (0.00) 23	£0.00 (£0.00)
Other outpatient appointment	0.00 (0.00) 22	£0.00 (£0.00)	0.00 (0.00) 23	£0.00 (£0.00)
Secondary Care Total	0.95 (1.00) 22	£934	1.00 (1.48) 23	£1,387
Social worker	0.45 (1.41) 22	£35.55 (£111.39)	0.00 (0.00) 23	£0.00 (£0.00)
Home care /home help	4.41 (20.68) 22	£52.92 (£247.20)	3.48 (11.01) 23	£41.76 (£132.00)
Day care	0.00 (0.00) 22	£0.00 (£0.00)	6.26 (20.74) 23	£287.96 (£952.20)
Drop in club	0.00 (0.00) 22	£0.00 (£0.00)	0.00 (0.00) 23	£0.00 (£0.00)
Other day care service	0.00 (0.00) 22	£0.00 (£0.00)	0.00 (0.00) 23	£0.00 (£0.00)
Social Care Total	4.86 (20.85) 22	£88	9.74 (22.49) 23	£330
Voluntary agency visit	0.00 (0.00) 22	£0.00 (£0.00)	0.09 (0.42) 23	£0.90 (£4.20)
Other primary or community based service	0.00 (0.00) 22	£0.00 (£0.00)	0.16 (0.80) 23	£3.54 (£17.69)
All Health & Social Care Visits Total	18.18	£1,484	20.20	£2,036
	% prescribed	Cost per person	% prescribed	Cost per person

For peer review only - http://bmjopen.bmj.com/site/about/guidelines.xhtml

	Mean, N	mean	Mean, N	mean
**Medications**				
Angiotensin II receptor antagonist	29% 25	£4.38	28%, 25	£4.23
ACE inhibitor	44%, 25	£3.04	48%, 25	£3.31
Aldosterone receptor antangonist	16%, 25	£10.09	24%, 25	£15.13
Anti-coagulant	15%, 25	£1.25	53%, 25	£4.42
Beta-blocker	56%, 25	£3.43	44%, 25	£2.69
Digoxin	8%., 25	£1.44	12%, 25	£2.16
Ivabradine	4%, 25	£10.33	4%, 25	£10.33
Loop diuretic	77%, 25	£6.14	76%, 25	£6.06
Nitrate	39%, 25	£108.15	19%, 25	£52.69
Thiazide diuretic	5%, 25	£0.48	1%, 25	£0.10
All Medications Total		£149		£101
All Health & Social Care Total		£1,632		£2,137
**Informal care**				
Caregiver hours per week	3.03 (5.86) 22	£72.72 (£140.64)	12.41 (30.30) 23	£297.60 (£727.20)
Non-caregiver hours per	4.98 (12.57) 22	£119.52 (£301.68)	0.46 (1.31) 23	£11.04 (£31.44)

For peer review only - http://bmjopen.bmj.com/site/about/guidelines.xhtml

week				
Total caring hours per week	8.01	£192	12.86	£309
Total caring hours per 6 months	208	£4,998.24	334	£8,025
Caregiver days off work	0.14 (0.64) 22	£17.12 (£78.28)	1.00 (4.38) 23	£122.31 (£535.71)
Non-caregiver days off work	0.14 (0.64) 22	17.12 (£78.28)	0.00 (0.00) 13	£0.00 (£0.00)
Total days off work (6-mths)	0.28	£34.25	1	£122.31
Patient days off work	0.00 (0.00) 22	£0.00 (£0.00)	0.00 (0.00) 23	£0.00 (£0.00)
Informal Care Total		£5,032		£8,147
**All Health, Informal & Social Care Total**		**£6,665**		**£10,284**

For peer review only

For peer review only - http://bmjopen.bmj.com/site/about/guidelines.xhtml

CONSORT 2010 checklist of information to include when reporting a randomised trial*

Section/Topic	Item No	Checklist item	Reported on page No
**Title and abstract**			
	1a	Identification as a randomised trial in the title	1
	1b	Structured summary of trial design, methods, results, and conclusions (for specific guidance see CONSORT for abstracts)	3
**Introduction**			
Background and objectives	2a	Scientific background and explanation of rationale	4
	2b	Specific objectives or hypotheses	5
**Methods**			
Trial design	3a	Description of trial design (such as parallel, factorial) including allocation ratio	6
	3b	Important changes to methods after trial commencement (such as eligibility criteria), with reasons	n/a
Participants	4a	Eligibility criteria for participants	6
	4b	Settings and locations where the data were collected	6
Interventions	5	The interventions for each group with sufficient details to allow replication, including how and when they were actually administered	7
Outcomes	6a	Completely defined pre-specified primary and secondary outcome measures, including how and when they were assessed	8
	6b	Any changes to trial outcomes after the trial commenced, with reasons	n/a
Sample size	7a	How sample size was determined	8
	7b	When applicable, explanation of any interim analyses and stopping guidelines	n/a
Randomisation:			
Sequence generation	8a	Method used to generate the random allocation sequence	6
	8b	Type of randomisation; details of any restriction (such as blocking and block size)	6
Allocation concealment mechanism	9	Mechanism used to implement the random allocation sequence (such as sequentially numbered containers), describing any steps taken to conceal the sequence until interventions were assigned	n/a
Implementation	10	Who generated the random allocation sequence, who enrolled participants, and who assigned participants to interventions	n/a
Blinding	11a	If done, who was blinded after assignment to interventions (for example, participants, care providers, those	6

For peer review only

For peer review only - http://bmjopen.bmj.com/site/about/guidelines.xhtml

		assessing outcomes) and how	
	11b	If relevant, description of the similarity of interventions	n/a
Statistical methods	12a	Statistical methods used to compare groups for primary and secondary outcomes	8-9
	12b	Methods for additional analyses, such as subgroup analyses and adjusted analyses	8-9
**Results**			
Participant flow (a diagram is strongly recommended)	13a	For each group, the numbers of participants who were randomly assigned, received intended treatment, and were analysed for the primary outcome	10
	13b	For each group, losses and exclusions after randomisation, together with reasons	10
Recruitment	14a	Dates defining the periods of recruitment and follow-up	10
	14b	Why the trial ended or was stopped	n/a
Baseline data	15	A table showing baseline demographic and clinical characteristics for each group	11-13
Numbers analysed	16	For each group, number of participants (denominator) included in each analysis and whether the analysis was by original assigned groups	11-13
Outcomes and estimation	17a	For each primary and secondary outcome, results for each group, and the estimated effect size and its precision (such as 95% confidence interval)	15
	17b	For binary outcomes, presentation of both absolute and relative effect sizes is recommended	18-24
Ancillary analyses	18	Results of any other analyses performed, including subgroup analyses and adjusted analyses, distinguishing pre-specified from exploratory	18-24
Harms	19	All important harms or unintended effects in each group (for specific guidance see CONSORT for harms)	18-24
**Discussion**			
Limitations	20	Trial limitations, addressing sources of potential bias, imprecision, and, if relevant, multiplicity of analyses	26
Generalisability	21	Generalisability (external validity, applicability) of the trial findings	26
Interpretation	22	Interpretation consistent with results, balancing benefits and harms, and considering other relevant evidence	26
**Other information**			
Registration	23	Registration number and name of trial registry	1 + 6
Protocol	24	Where the full trial protocol can be accessed, if available	6
Funding	25	Sources of funding and other support (such as supply of drugs), role of funders	28

*We strongly recommend reading this statement in conjunction with the CONSORT 2010 Explanation and Elaboration for important clarifications on all the items. If relevant, we also recommend reading CONSORT extensions for cluster randomised trials, non-inferiority and equivalence trials, non-pharmacological treatments, herbal interventions, and pragmatic trials. Additional extensions are forthcoming: for those and for up to date references relevant to this checklist, see www.consort-statement.org.

For peer review only
For peer review only - http://bmjopen.bmj.com/site/about/guidelines.xhtml

BMJ Open

A Randomised Controlled Trial of a Facilitated Home-Based Rehabilitation Intervention in Patients with Heart Failure with Preserved Ejection Fraction and their Caregivers: REACH-HFpEF Pilot Study.

Journal:	*BMJ Open*
Manuscript ID	bmjopen-2017-019649.R1
Article Type:	Research
Date Submitted by the Author:	06-Feb-2018

Complete List of Authors:	Lang, Chim C.; Univ Dundee, Division of Molecular & Clinical Medicine<br>Smith, Karen; University of Dundee, Molecular & Clinical Medicine/School of Nursing<br>Wingham, Jennifer; Royal Cornwall Hospitals NHS Trust, Research, Development and Innovation; University of Exeter Medical School,<br>Eyre, Victoria; Plymouth University, Peninsula Clinical Trials Unit<br>Greaves, Colin; University of Exeter Medical School, Institute of Health Research<br>Warren, Fiona; University of Exeter Medical School, Institute of Health Research<br>Green, Colin; University of Exeter Medical School, Institute of Health Research<br>Jolly, Kate; University of Birmingham<br>Davis, Russell; Sandwell & West Birmingham Hospitals NHS Trust, Dept of Cardiology<br>Doherty, Patrick Joseph; University of York, Health Science<br>Miles, Jackie; Anuerin Bevan University Health Board, Research and Development<br>Britten, Nicky; University of Exeter Medical School, PenCLAHRC: National Institute for Health Research (NIHR) Collaboration for Leadership in Applied Health Research and Care (CLAHRC) for the South West Peninsula; University of Exeter Medical School, Institute for Health Service Research<br>Abraham, Charles; University of Exeter Medical School, Psychology Applied to Health Group<br>Van Lingen, Robin; Royal Cornwall Hospitals NHS Trust, Cardiology<br>Singh, Sally; University Hospitals of Leicester NHS Trust, Cardiac/Pulmonary Rehabilitation<br>Paul, Kevin; Royal Cornwall Hospitals NHS Trust, REACH-HF Patient and Public Involvement Group, c/o Research, Development & Innovation<br>Hillsdon, Melvyn; University of Exeter<br>Sadler, Susannah; University of Exeter Medical School, Institute of Health Research<br>Hayward, Christopher; Plymouth University, Peninsula Clinical Trials Unit<br>Dalal, Hayes; Royal Cornwall Hospitals NHS Trust, Teaching and Research<br>Taylor, Rod; University of Exeter, Peninsula Medical School

**Primary Subject Heading**:	Cardiovascular medicine

Secondary Subject Heading:	Rehabilitation medicine
Keywords:	Heart Failure,, Heart Failure with Preserved Ejection Fraction, Cardiac Rehabilitation, Randomised Controlled Trial, Complex Intervention

SCHOLARONE™ Manuscripts

For peer review only

A Randomised Controlled Trial of a Facilitated Home-Based Rehabilitation Intervention in Patients with Heart Failure with Preserved Ejection Fraction and their Caregivers: REACH-HFpEF Pilot Study

Corresponding Author:

Chim Choy Lang,
Division of Molecular and Clinical Medicine
School of Medicine
Mailbox 2
Ninewells Hospital & Medical School
Dundee
DD1 9SY
E: c.c.lang@dundee.ac.uk
T: 01382 383013

ISRCTN 57596739

Lang Chim C (Professor/Medical Consultant)[1], Smith Karen (Nurse Consultant)[1,2], Wingham Jenny (Honorary Clinical Senior Lecturer/Senior Clinical Researcher)[3,4], Eyre Victoria (Centre Manager)[5], Greaves Colin J (Associate Professor)[3], Warren Fiona C (Senior Lecturer)[3], Green Colin (Professor)[3], Jolly Kate (Professor) [6], Davis Russell C (Medical Consultant)[7], Doherty Patrick (Professor)[8], Miles Jackie (Lead Nurse Heart Failure/Cardiac Rehabilitation Research)[9], Britten Nicholas (Professor)[3], Abraham Charles (Professor)[3,] , Van Lingen Robin (Medical Consultant)[10], Singh Sally (Professor)[11], Paul Kevin (Patient Representative)[12], Hillsdon Melvyn (Professor)[13] , Sadler Susannah (Research Fellow)[3], Hayward Christopher (Senior Trial Manager)[14], Dalal Hayes M (General Practitioner/Honorary Clinical Associate Professor)[3,4], Taylor Rod S (Professor)[3] and on behalf of the REACH-HF investigators

[1]School of Medicine, University of Dundee, Ninewells Hospital and Medical School, Dundee, UK

[2]School of Nursing & Health Sciences, University of Dundee, Dundee, UK

[3]Institute of Health Research, University of Exeter Medical School, Exeter, UK

[4]Research, Development & Innovation, Royal Cornwall Hospitals NHS Trust, Truro, UK

[5]Re:Cognition Health, Plymouth, UK

[6]Institute for Applied Health Research, University of Birmingham, Birmingham, UK

For peer review only - http://bmjopen.bmj.com/site/about/guidelines.xhtml

[7]Cardiology Department, Sandwell & West Birmingham Hospitals NHS Trust, Birmingham, UK

[8]Department of Health Sciences, University of York, York, UK

[9]Research and Development, Anuerin Bevan University Health Board, St Woolos Hospital, Newport, UK

[10]Cardiology Department, Royal Cornwall Hospitals NHS Trust, Truro, UK

[11]Centre for Exercise and Rehabilitation Science, University Hospitals of Leicester NHS Trust, Glenfield Hospital, Leicester, UK

[12]REACH-HF Patient and Public Involvement Group, c/o Research, Development & Innovation, Royal Cornwall Hospitals NHS Trust, Truro, UK

[13]Sport and Health Sciences, University of Exeter, Exeter, UK

[14]Peninsula Clinical Trials Unit, University of Plymouth, Plymouth, UK

Word count: 3657; abstract – 290

For peer review only

For peer review only - http://bmjopen.bmj.com/site/about/guidelines.xhtml

Abstract

Introduction

Home-based cardiac rehabilitation may overcome suboptimal rates of participation. The overarching aim of this study was to assess the feasibility and acceptability of the novel REACH-HF rehabilitation intervention for patients with heart failure with preserved ejection fraction (HFpEF) and their caregivers.

Methods and results

Patients were randomised 1:1 to REACH-HF intervention plus usual care (intervention group) or usual care alone (control group). REACH-HF is a home-based comprehensive self-management rehabilitation programme that comprises patient and carer manuals with supplementary tools, delivered by trained healthcare facilitators over a 12-week period. Patient outcomes were collected by blinded assessors at baseline, 3 and 6 months post-randomisation and included health-related quality of life (primary) and psychological well-being, exercise capacity, physical activity and HF-related hospitalisation (secondary). Outcomes were also collected in caregivers.

We enrolled 50 symptomatic heart failure patients from Tayside, Scotland with a left ventricular ejection fraction ≥ 45% (mean age: 73.9 years, 54% female, 100% white British) and 21 caregivers. Study retention (90%) and intervention uptake (92%) were excellent. At 6 months, data from 45 patients showed a potential direction of effect in favour of the intervention group, including the primary outcome of Minnesota Living with Heart Failure Questionnaire total score (between group mean difference: -11.5, 95% confidence interval: -22.8 to 0.3). A total of 11 (4 intervention, 7 control) patients experienced a hospital admission over the 6 months follow up with 4 (control patients) of these admissions being HF-related. Improvements were seen in a number intervention caregiver mental health and burden compared to control.

Conclusions

Our findings support the feasibility and rationale for delivering the REACH-HF facilitated home-based rehabilitation intervention for patients with HFpEF and their caregivers and progression to a full multicentre randomised clinical trial to test its clinical and cost-effectiveness.

Key words: Heart Failure, Heart Failure with Preserved Ejection Fraction, Cardiac Rehabilitation, Randomised Controlled Trial, Complex Intervention, Caregivers.

Trial registration number: ISRCTN78539530

For peer review only - http://bmjopen.bmj.com/site/about/guidelines.xhtml

Strengths

- REACH-HF is the first comprehensive home-based, self-management cardiac rehabilitation intervention for HFpEF patients and their caregivers.
- The findings of this pilot study support the feasibility and acceptability of the home-based REACH-HF rehabilitation intervention in patients with HFpEF and their caregivers and indicate that it is feasible to recruit and retain participants in a randomised trial with follow-up.
- Potential favourable impacts of the REACH-HF intervention on caregiver mental health and measures of burden were observed in this pilot study.

Limitations

- This study was not designed or powered to definitively assess the efficacy or safety of the REACH-HF intervention in HFpEF.
- Generalisability of this study's findings is limited, given it was based in a single centre.

Introduction

Epidemiological data show that approximately half of those patients with clinical features of heart failure (HF) have preserved ejection fraction (HFpEF).[1] In contrast to HF with reduced ejection fraction (HFrEF), the prevalence of HFpEF is increasing.[2] Importantly, the substantial burden from HFpEF appears to be similar to HFrEF, measured by exercise intolerance, poor health-related quality of life (HRQoL), mortality, increased hospital admissions and higher healthcare costs.[3] Although drug and device therapy have helped to improve outcomes in HFrEF, prognosis in HFpEF remains unchanged, with no large-scale randomised trial demonstrating significant treatment benefits that alter the natural course of HFpEF or lower mortality.[4,5] However, systematic reviews and meta-analyses have shown promising evidence for the benefit of exercise-based cardiac rehabilitation (CR) in HFpEF.[6,7] A recent meta-analysis of 8 randomised trials in 317 HFpEF patients found exercise-based CR significantly improved exercise capacity and HRQoL compared to usual care.[7] The

For peer review only - http://bmjopen.bmj.com/site/about/guidelines.xhtml

CR programmes undertaken in these trials were predominantly group-based, supervised, and delivered in centre-based settings.

Participation of patients with HF in CR remains suboptimal.[8,9] A United Kingdom survey found that only 16% of CR centres provided a HF programme; commonly cited reasons for the lack of CR provision were a lack of resources and exclusion from commissioning agreements.[9] Two main reasons given by patients for failing to take part in CR are difficulties with regular attendance at their local hospital centre and reluctance to join group-based classes.[9]

There is increasing recognition of the possibility of alternative delivery models of CR, such as home-based programmes, in order to overcome suboptimal rates of CR uptake seen with HF.[10,11] Facilitated home-based CR has been shown to provide similar benefits to centre-based CR in terms of clinical and HRQoL outcomes at equivalent cost for those with HF and following myocardial infarction and revascularization.[11,12]

The Rehabilitation EnAblement in CHronic Heart Failure (REACH-HF) programme of research was designed to develop and evaluate a home-based comprehensive self-management rehabilitation intervention, including a self-care manual, an exercise programme, and facilitation by health professionals designed to improve self-management and HRQoL in people with HF. [13,14] In addition to REACH-HF intervention includes a 'Family and Friends Resource' designed to support caregivers.

The overarching aim of this study was to assess the feasibility of undertaking definitive randomised trial to assess the clinical effectiveness and cost-effectiveness of the REACH-HF intervention in patients with HFpEF and their caregivers. Specific objectives of the study were to: (1) assess the acceptability of the study design and procedures to participants (patients and caregivers); (2) assess feasibility and experience of the delivery of intervention for participants and healthcare professional facilitators; (3) identify barriers to participation in the intervention and study procedures; (4) inform a definitive study sample size; (5) assess methods for the collection of data including resource use and costs; and (6) assess the fidelity of the delivery of the REACH-HF intervention by healthcare professional facilitators.

For peer review only - http://bmjopen.bmj.com/site/about/guidelines.xhtml

Methods

The study design and methods have been described in the published study protocol.[14] The study is reported in accordance with the Consolidated Standards of Reporting Trials (CONSORT) extension for pilot trials.[15]

Design

The REACH-HFpEF pilot study was a single centre (Tayside, Scotland) two group randomised controlled trial with a parallel mixed methods feasibility evaluation and assessment of costs. Participants were individually randomised in a 1:1 ratio to the REACH-HF intervention plus usual care (intervention group) or usual care alone (control group). Given the nature of the REACH-HF intervention, it was not possible to blind participants or those involved in the provision of care. However, the statistician (FCW) undertaking the data analysis was blinded to treatment allocation and we also blinded researchers undertaking collection of outcome data to minimise potential bias. We assessed the fidelity of blinding by asking outcome assessors at each follow-up visit to guess patient group allocation. Unblinding of groups did not take place until after data analysis and the blinded results had been presented to the Trial Management Group and interpretation of results was agreed. Approvals were obtained from Scotland A Research Ethics Committee and the study was registered (ISRCTN 57596739).

Study population

The study population included patients and their caregivers. Participating patients were aged 18 years or older and had a confirmed diagnosis of HFpEF on echocardiography, radionuclide ventriculography or angiography (i.e. left ventricular ejection fraction ≥ 45% within the last 6 months prior to randomisation). Patients who had undertaken CR within 6 months prior to enrolment were excluded, as were patients with a contraindication to exercise testing or exercise training (with consideration of adapted European Society of Cardiology guidelines for HF).[14,16] Participating caregivers were aged 18 years or older and provided unpaid support to participating. Patients who did not have an identified caregiver were able to participate, as were those whose caregiver was not willing to participate in the study.

For peer review only - http://bmjopen.bmj.com/site/about/guidelines.xhtml

Intervention

The REACH-HF intervention is described in detail elsewhere[17] In summary, REACH-HF is a comprehensive self-management programme informed by evidence, theory, and service user perspective. It comprises the 'Heart Failure Manual' (REACH-HF Manual), Relaxation CD, chair-based exercise DVD, a 'Progress Tracker' tool for patients, and a 'Family and Friends Resource' for caregivers. Participating patients and caregivers worked through the REACH-HF Manual over a 12-week period with facilitation by two trained cardiac nurses. The facilitators provided support as needed of which at least one was face-to-face and two were by telephone contacts. The REACH-HF manual incorporates 5 core informative and interactive elements covering a wide range of topics relating to living with/adapting to living with HF, and includes:

1. a progressive exercise training programme, tailored according to initial fitness assessments, delivered as a walking programme or a chair-based exercise DVD, or a combination of the two (as selected by the patient);
2. managing stress/breathlessness/anxiety;
3. HF symptom monitoring;
4. taking medication; and
5. understanding HF (and why self-management helps).

The REACH-HF Manual was designed for patients with HFrEF (in terms of coverage of medication and explanations of condition). There was limited evidence to guide the development of the REACH-HF Manual for HFpEF patients. Thus it was adapted for this pilot study to allow evaluation in patients with HFpEF. The majority of the self-management advice in all other sections of the REACH-HF Manual is relevant to all patients with HF and corresponds to national HF guidelines.[18,19] The core priorities for caregiver elements of the intervention were:

1. To facilitate improvement in patient HRQoL by helping them to achieve the core priorities for change.
2. To improve HRQoL for caregivers by acting to maintain their own health and well-being.

Usual care

Both intervention and control group patients received usual medical management for HF according to current guidelines.[18,19]

For peer review only - http://bmjopen.bmj.com/site/about/guidelines.xhtml

Outcome measures and follow up

We collected the following pilot study outcomes: recruitment rate for participants (patients and caregivers) across the various recruitment pathways; attrition and loss to follow up; completeness of participant outcome measures at follow up; fidelity of the REACH-HF Manual delivery by intervention facilitators (sample of patient-facilitator contacts for sample of 6 patients were audio recorded and independently reviewed using a 13-item checklist (developed by CJG and JW) by two researchers (KS and Karen Coyle)); acceptability of the intervention (via face-to-face semi-structured interviews with purposive sample of 15 patients, 7 caregivers and both facilitators at the end of the intervention delivery period); and acceptability of study participation to participants (via interviews and questionnaire).

The following participant outcomes proposed for a future definitive trial were collected at baseline (pre-randomisation) and follow up at 4 and 6 months post randomisation:

Patients - disease-specific HRQoL (Minnesota Living with Heart Failure questionnaire (MLHFQ) (primary outcome);[20] and Heart Related Quality of Life (HeartQoL) questionnaire);[21] clinical events (all-cause mortality, hospital admission related to HF and not related to HF (relatedness was independently adjudicated by a panel of 3 cardiologists); exercise capacity (incremental shuttle walking test ISWT);[22] physical activity level (GeneActive accelerometry over a 7-day period);[23] psychological wellbeing (Hospital Anxiety and Depression Scale questionnaire, HADS);[24] generic HRQoL (EQ-5D-5L questionnaire);[25] Self-care of HF Index questionnaire (SCHFI);[26] healthcare utilisation (primary and secondary care contacts, social care contacts and relevant medication usage, reported by patient participants); and safety outcomes (serious adverse events).

Caregivers - Caregiver Burden Questionnaire-HF (CBQ-HF),[27] Caregiver Contribution to Self-care of HF Index questionnaire (CC-SCHFI);[26] Family Caregiver Quality of Life Scale questionnaire (FAMQOL);[28] Generic HRQoL (EQ-5D-5L);[25] and psychological wellbeing (HADS).[24]

Data analysis

Our planned recruitment target of 50 patients allowed us to achieve the feasibility aims and objectives of this study, i.e., an estimate of attrition, estimates of the

For peer review only - http://bmjopen.bmj.com/site/about/guidelines.xhtml

standard deviation (SD) of the primary and secondary outcomes to inform power for a future definitive trial, and sufficient numbers for qualitative interviews.

We report the mean and SD (or relevant summary statistics) for both groups for all patient and caregiver outcomes at each follow-up point and the mean (and 95% confidence interval (CIs)) for the between group difference in outcomes at 6-month follow-up using linear regression models adjusting for baseline outcome. Given the pilot nature of this trial, we do not report p-values for the comparison of outcomes between groups. All analyses are based on the intention to treat principle (patients are analysed according to their original random allocation) using observed data only.

Data on patient resource use related to health and social care were collected using a standardised resource use questionnaire at baseline (for previous 6 months) and at 4 and 6 months follow-up. Unit costs per item of resource use were obtained from published estimates and where necessary inflated to 2016 prices using the Healthcare and Community Health Services index (see eTable 1).[29] These unit costs were then applied to the resource use reported at patient level to estimate the delivery costs associated with the REACH-HF manual, and the total costs associated with health and social care at baseline and over the 6-month follow-up. As with clinical outcome, costs are presented descriptively. EQ-5D-3L utilities were obtained using existing crosswalk values from EQ-5D-5L.[30] All outcomes and costs analyses were conducted using Stata (v14.2; College Station, TX, USA: StataCorp LP).

Patient, caregiver, and facilitator interviews were transcribed verbatim and analysed using thematic analysis and will be fully reported elsewhere.[31]

For peer review only

For peer review only - http://bmjopen.bmj.com/site/about/guidelines.xhtml

Results

Recruitment and retention of patients and caregivers and acceptability of trial design

Study enrolment, allocation, and follow-up of study participants are summarised in the CONSORT flow diagram shown in Figure 1. Between April 2015 and June 2016, 225 potential patients were approached and 50 were randomised (intervention group: 25; control group: 25) i.e. 22% (95% CI: 17% to 28%) of patients approached. The original forecast was a recruitment rate of 5 patients per month. However, the actual recruitment rate during the trial was 4.5 patients per month, resulting in a 1-month extension to the period of recruitment. A caregiver was recruited in connection with 21 (42%) patient participants (intervention group: 11; control group: 10).
At 6-month follow-up, 5 out 50 (10%, 95%: 3% to 22%) patients were lost to follow-up. Seventeen out of the 21 recruited caregivers provided follow up data at 6-months. Patients and caregivers rated a high level of satisfaction with their participation in the trial (see eTable 2).

Baseline characteristics of patients and caregivers

There was evidence of imbalance between intervention and control group patients in terms of their baseline demographic characteristics (see Table 1). Compared with the control group, the intervention group included a higher proportion of females, and lower proportions of patients with an ischaemic diagnosis, with atrial flutter/atrial fibrillation, and with chronic renal failure; also, the intervention group had a younger mean age. Caregivers were typically the partner or children of patients, were of a younger mean age than participating patients and predominantly female.

Table 1a Patient baseline demographic characteristics
Table 1b Caregiver baseline demographic characteristics

For peer review only

For peer review only - http://bmjopen.bmj.com/site/about/guidelines.xhtml

Table 1a. Patient baseline demographic characteristics

	**Intervention**<br>N = 25<br>Mean (SD) or N (percent)	**Control**<br>N = 25<br>Mean (SD) or N (percent)
Gender: male	9 (36)	14 (56)
Age (years)	71.8 (9.9)	76.0 (6.6)
BMI (kg^2/m)	32.1 (6.3)	32.2 (5.3)
Ethnic group: white	25 (100)	25 (100)
Relationship status:		
Single	4 (16)	2 (8)
Married	14 (56)	8 (32)
Divorced/civil partnership dissolved	1 (4)	3 (12)
Widowed	6 (24)	12 (48)
Domestic residence:		
Live alone	9 (36)	14 (56)
Spouse/partner only	14 (56)	8 (32)
Spouse/partner & child > 18 years	0 (0)	2 (8)
Other adult family members only	2 (8)	1 (4)
Smoking status:		
Never smoked	2 (8)	2 (8)
Ex-smoker	15 (60)	14 (56)
Current smoker	8 (32)	9 (36)
NYHA status:		
Class I	1 (4)	1 (4)
Class II	15 (60)	16 (64)
Class III	9 (36)	8 (32)
Class IV	0 (0)	0 (0)

For peer review only - http://bmjopen.bmj.com/site/about/guidelines.xhtml

For peer review only

Cause of heart failure:[1]		
Ischaemic	8 (32)	16 (64)
Non-ischaemic	16 (64)	8 (32)
Unknown	1 (4)	1 (4)
Number of comorbidities:		
0	7 (28)	12 (48)
1	15 (60)	6 (24)
2	3 (12)	4 (16)
3	0	2 (8)
4	0	1 (4)
Previous myocardial infarction	4 (16)	5 (20)
Previous atrial fibrillation/atrial flutter	6 (24)	13 (52)
Hypertension	18 (72)	14 (56)
Diabetes mellitus	9 (36)	6 (24)
Chronic renal impairment	3 (13)	10 (40)
Time since diagnosis of HF (years):		
< 1	6 (24)	4 (16)
1 to 2	7 (28)	6 (24)
> 2	12 (48)	15 (60)
Medication:		
Beta-blocker	18 (72)	13 (52)
Angiotensin 2 receptor antagonist	7 (28)	7 (28)
ACE inhibitor	11 (44)	14 (56)
Main activity:		
In employment or self-employment	0 (0)	1 (4)
Retired	22 (88)	24 (96)
Unemployed	2 (8)	0 (0)
Other	1 (4)	0 (0)

For peer review only - http://bmjopen.bmj.com/site/about/guidelines.xhtml

For peer review only

Education:		
Post school	7 (28)	7 (28)
Degree	5 (20)	5 (20
Pro-BNP levels:		
≤ 2000 pg/ml	23 (92)	22 (88)
> 2000 pg/ml	2 (8)	3 (12)

[1]Cause of HF determined by Principal Investigator

Table 1b. Caregiver baseline demographic characteristics

	**Intervention** N=11[1] Mean (SD) or N (percent)	**Control** N=10 Mean (SD) or N (percent)
Gender: male	3 (30)	2 (20)
Age (years)	59.3 (14.0)	64.8 (11.6)
Relationship to patient		
Partner	4 (40)	6 (60)
Son/daughter	3 (30)	4 (40)
Sibling	2 (20)	0 (0)
Friend	1 (10)	0 (0)

[1]One caregiver withdrew shortly after randomisation and did not provide baseline da

Completion of outcome measures by patients and caregivers and fidelity of blinding by outcome assessors

We collected data from 45/50 patients (90%, 95% CI: 78% to 97%) at 6-month follow-up on the MLHFQ, our proposed primary outcome. Levels of completion of patient secondary outcomes and caregiver outcomes were consistently high (≥ 76% of participants for all outcomes). The one exception was the ISWT, which had notably lower level of completion (35 (78%) patients at 4-month follow-up and 33 (73%) patients at 6-month follow-up).

Outcome assessors correctly guessed patient group allocation in 22% of cases (10/45) at 4 months and 20% of cases (19/45) at 6 months, indicating that blinding was likely to have been maintained.

For peer review only

For peer review only - http://bmjopen.bmj.com/site/about/guidelines.xhtml

Acceptability of patient, carers and facilitators of REACH-HF intervention and fidelity of intervention delivery by facilitators

Qualitative interviews and observations of the patient and caregivers interactions with the facilitator indicated high levels of satisfaction, acceptability and the feasibility of delivering the REACH HF Intervention in HFpEF patients (see eTable 3). One of the most highly valued elements of the REACH-HF by participants was the role of the facilitator, who was seen to act as an educator, a source of emotional support and reassurance as well as a motivator and enabler.

Of the 6 patients selected for inclusion, a total of ~45 hours of patient-facilitator interaction was used for analysis. Fidelity scoring indicated adequate delivery (defined as a score of 3 or more) for most aspects of the intervention by the two facilitators (see eTable 4). Of the 6 patients selected for inclusion, a total of ~45 hours of patient-facilitator interaction was used for analysis. Mean score for items 9 (addressing emotional consequences of being a caregiver) and 11 (caregiver health and well-being) was less than 3.

Patient adherence to REACH-HF intervention

Twenty three of the 25 (92%) intervention patients met our minimum adherence criteria of attendance i.e. attendance at the first face-to-face meeting with the facilitator and at least two further facilitator contacts (either face-to-face or telephone). In these patients, the mean number of facilitator contacts was 6.2 (SD: 1.6), the majority of which were face-to-face contacts (mean 5.1; SD: 1.5) and the remainder were telephone contacts (mean: 1.1, SD: 1.3) (see Table 2).

Table 2 REACH-HF intervention delivery: healthcare resource use and costs

For peer review only - http://bmjopen.bmj.com/site/about/guidelines.xhtml

Table 2. REACH-HF intervention delivery: healthcare resource use and costs

	**Number of patient contacts** Mean (SD)	**Duration of patient contacts contact (minutes)** Mean (SD)	**Duration facilitator non-contact planning (minutes)** Mean (SD)	**Duration facilitator travel (minutes)** Mean (SD)
Face to face contacts/patient	5.1 (1.5)	60.6 (29.6)	17.2 (24.4)	40.2 (37.4)
Telephone contacts/patient	1.1 (1.3)	7.7 (4.0)	8.0 (9.5)	
Total contacts/patients	6.2 (1.6)			
Total time, face to face contacts		308.9 (123.3)		
Total time, telephone contacts		8.8 (10.3)		
Total facilitator planning/non-contact time, face to face, minutes		87.4 (55.8)		
Total facilitator planning/non-contact time, telephone, minutes		9.1 (12.6)		
Overall total time input, time		414.2 (145.4)		
		**Cost per patient**[1] Mean (SD)		
Estimated total HF Facilitator cost,		£303.64 (£106.59)		

For peer review only - http://bmjopen.bmj.com/site/about/guidelines.xhtml

Other resource use/costs:<br>- consumables (1 x manual)<br>- DVDs (x 2, at £7.50 each)<br>- Distribution of HF Facilitator training costs, per participant[2]		 £25.00<br>£15.00<br>£18.97		
Estimated total delivery cost of HF-REACH intervention		£362.61		

[1]Unit costs – Staff: Staff grade equivalent to 'Community Nurse' (includes district nursing sister, district nurse) and Nurse Specialist (community), from Curtis and Burns, Unit Costs of Health and Social Care 2016, p141-142. Based on Agenda for Change band 6 (staff salary at £32,114 pa). Estimated cost per hour = £44 (Curtis and Burns, 2016); Includes salary, salary on-costs, overheads (management costs, and non-staff costs (including travel/transport]), capital overheads, and excludes costs for qualifications.

[2]Training cost per REACH HF Facilitator, specific to delivery of the REACH-HF intervention, are estimated at £1,897 (involving 3 days, i.e. 24 hours training at £44/hour; costs for Trainer/s per Trainee at £366, assuming 8 Trainees per 3 day course, and Trainers at Agenda for Change, Band 8a, £61/hr (Curtis and Burns, 2016); cost for REACH-HF Facilitator Manual at £400 each; plus estimate of consumables for training sessions). These costs are distributed across the first 100 participants/patients receiving the intervention, resulting in an estimate of £18.97 per participant.

Participant outcomes

Patients

Patient outcome results at baseline, and 3-month and 6-month follow-up, and between group differences at 6-month follow-up are shown in Table 3 (see eTable 5 for baseline-follow up within group changes). At 6 months, a number of patient outcomes potential direction of effect in favour of intervention, including MLWHF total score (Figure 2) (between group mean difference: -11.5, 95% confidence interval (CI): -22.8 to 0.3), HeartQoL global score (0.5, 95% CI: 0.0 to 0.9), EQ-5D-3L utility index (0.11, 95% CI: -0.04 to 0.26), HADS depression score (-1.5, 95% CI: -3.4 to 0.3), and SCHFI maintenance score (9.5, 95% CI: 2.5 to 17.3). The direction of possible intervention effects were less clear for the outcomes of ISWT and level of physical activity.

For peer review only - http://bmjopen.bmj.com/site/about/guidelines.xhtml

At 6-months follow up, 11 (4 intervention, 7 control) patients experienced a hospital admission with 4 (all control) of these admissions being HF-related. All these serious adverse events were considered to be unrelated to the study processes or to the REACH-HF intervention. One control patient died related to HF shortly after the 6-month follow-up.

Table 3 Patient outcomes at baseline and follow-up

For peer review only

For peer review only - http://bmjopen.bmj.com/site/about/guidelines.xhtml

Table 3. Patient outcomes at baseline and follow-up

	**Baseline**		**4-month follow-up**		**6-month follow-up**		
	**Intervention** Mean (SD), N	**Control** Mean (SD), N	**Intervention** Mean (SD), N	**Control**, Mean (SD), N	**Intervention** Mean (SD), N	**Control** Mean (SD,) N	**Mean between group difference[1] (95% CI)**
Primary outcome							
MLHFQ, Overall	38.2 (27.6), 25	36.0 (26.5), 25	35.5 (28.3), 22	37.8 (27.9), 23	29.2 (25.8), 22	38.7 (30.1), 23	-11.5 (-22.8 to 0.3)
MLHFQ, Physical	21.6 (13.4), 25	19.8 (12.4), 25	19.4 (13.5), 22	20.7 (12.8), 23	16.2 (12.3), 21	20.3 (13.6), 23	-4.7 (-10.1 to 0.8)
MLHFQ, Emotional	7.8 (9.1), 25	7.8 (8.4), 25	8.0 (8.5), 22	9.1 (8.6), 23	6.8 (8.1), 21	9.0 (8.5), 23	-2.7 (-6.0 to 0.6)
Secondary outcomes							
HADS, Anxiety	5.6 (4.8), 25	6.1 (4.9), 25	5.7 (4.8), 22	6.4 (5.4), 23	5.5 (5.1), 21	6.0 (5.1), 23	-0.2 (-2.6 to 2.1)
HADS, Depression	6.2 (4.2), 25	5.6 (4.1), 25	5.6 (4.4), 22	6.6 (4.5), 23	5.4 (4.3), 21	6.9 (5.2), 23	-1.5 (-3.4 to 0.3)
Heart-QoL, Global	1.4 (0.8), 25	1.6 (0.9), 25	1.5 (1.0), 22	1.4 (1.0), 23	1.8 (0.8), 21	1.4 (1.1), 23	0.5 (0.0 to 0.9)

For peer review only - http://bmjopen.bmj.com/site/about/guidelines.xhtml

Heart-QoL, Physical	1.2 (0.8), 25	1.4 (1.0), 25	1.3 (1.0), 22	1.3 (1.0), 23	1.6 (0.8), 21	1.3 (1.1), 23	0.5 (0.0 to 1.0)
Heart-QoL, Emotional	2.0 (1.0), 25	2.0 (1.0), 25	2.0 (1.0), 22	1.9 (1.0), 23	2.2 (1.0), 21	1.8 (1.1), 23	0.3 (-0.1 to 0.8)
EQ-5D-3L, index score	0.57 (0.29), 25	0.58 (0.31), 24	0.60 (0.28), 22	0.52 (0.34), 23	0.65 (0.31), 21	0.55 (0.29), 23	0.11 (-0.04 to 0.26)
SCHFI, Maintenance	51.9 (13.9), 25	45.3 (16.5), 25	68.9 (14.9), 22	49.6 (14.4), 23	64.2 (12.8), 21	48.9 (14.3), 23	9.9 (2.5 to 17.3)
SCHFI, Management	37.6 (20.7), 23	37.8 (18.4), 18	48.9 (26.5), 19	32.6 (19.2), 17	45.0 (2.7), 14	37.6 (23.5) 15	8.0 (-8.9 to 25.0)
SCHFI, Confidence	60.4 (25.5), 25	56.9 (23.0), 25	65.2 (18.7), 22	49.5 (24.9), 23	62.1 (20.0), 21	53.4 (26.1), 23	6.6 (-6.7 to 19.9)
ISWT (metres)	183.6 (174.2), 25	157.6 (117.8), 23	218.9 (185.5), 18	178.2 (115.0), 17	224.7 (161.4), 17	183.8 (98.1), 16	-2.1 (-39.4 to 35.2)
Accelerometry, number of days/week with at least 10 minutes/day activity > 100mg	5.8 (2.3), 25	5.9 (2.0), 25	5.6 (2.4), 21	5.7 (1.9), 21	4.9 (2.7), 19	6.0 (2.1), 20	-0.4 (-1.3 to 0.5)

For peer review only - http://bmjopen.bmj.com/site/about/guidelines.xhtml

Accelerometry, sverage time/day at ≤ 20mg (mins)	1126 (98), 25	1090 (112), 25	1115 (110), 21	1103 (124), 21	1136 (101), 19	1098 (114), 20	-10 (-49 to 28)
Accelerometry, average time/day at 21 to 40mg (mins)	128 (33), 25	152 (39), 25	140 (38), 21	143 (36), 21	134 (37), 19	148 (41), 20	12 (-4 to 29)
Accelerometry, average time/day at 41 to 60mg (mins)	77 (27), 25	87 (29), 25	79 (29), 21	84 (33), 21	75 (25), 19	85 (27), 20	1 (-10 to 12)
Accelerometry, average time/day at 61 to 80mg (mins)	45 (20), 25	47 (20), 25	45 (23), 21	45 (21), 21	40 (20), 19	45 (19), 20	-1 (-9 to 6)
Accelerometry, average time/day at 81 to 100mg (mins)	25 (14), 25	25 (15), 25	25 (15), 21	25 (17), 21	22 (15), 19	25 (15), 20	-1 (-6 to 4)

For peer review only - http://bmjopen.bmj.com/site/about/guidelines.xhtml

Accelerometry, average time/day at > 100mg (mins)	39 (30), 25	40 (48), 25	36 (31), 21	39 (52), 21	32 (30), 19	39 (48), 20	-2 (-9 to 5)

[1] Mean between group differences (intervention minus control) adjusted for baseline values.

For peer review only

For peer review only - http://bmjopen.bmj.com/site/about/guidelines.xhtml

Caregivers

Caregiver outcome results at baseline and 3-month and 6-month follow-up are shown in Table 4 (see eTable 6 for within group results). There were indications of a favourable intervention effect for some outcomes including HADS depression and anxiety scores and CBQ-HF emotional and CC-SCHFI maintenance domain scores.

Table 4 Caregiver outcomes at baseline and follow-up

For peer review only

For peer review only - http://bmjopen.bmj.com/site/about/guidelines.xhtml

Table 4. Caregiver outcomes at baseline and follow-up

	Baseline		4-month follow-up		6-month follow-up		
	**Intervention** Mean (SD), N	**Control,** Mean (SD), N	**Intervention,** Mean (SD), N	**Control,** Mean (SD), N	**Intervention,** Mean (SD), N	**Control,** Mean (SD), N	**Mean between group difference[1] (95% CI)**
HADS, Anxiety	8.6 (5.4), 10	6.2 (5.5), 10	7.1 (7.0), 8	6.8 (3.0), 10	6.3 (6.2), 8	7.6 (4.7), 9	-3.4 (-6.6 to 0.2)
HADS, Depression	4.0 (4.0), 10	4.7 (4.3), 10	3.9 (3.2), 8	5.4 (3.8), 10	2.9 (3.4), 8	5.9 (3.4), 9	-2.3 (-5.1 to -0.5)
FAMQOL, Overall	61.4 (10.5), 10	56.9 (12.0), 10	60.0 (10.2), 8	54.3 (12.6), 10	56.8 (8.6), 8	54.0 (8.7), 9	-1.1 (-7.9 to 5.6)
FAMQOL, Physical	17.0 (2.6), 10	14.9 (3.3), 10	15.9 (2.9) 8	14.9 (3.1), 10	15.8 (1.8), 8	15.0 (2.2), 9	-1.2 (-2.7 to 0.3)
FAMQOL, Psychological	13.9 (5.3), 10	13.5 (5.2), 10	13.3 (4.5), 8	12.0 (4.2), 10	12.8 (5.0), 8	12.1 (3.6), 9	0.3 (-2.7 to 3.3)
FAMQOL, Social	16.6 (2.8), 10	15.8 (4.7), 10	16.3 (2.4), 8	14.8 (3.6), 10	15.6 (0.9), 8	14.8 (2.5), 9	0.0 (-1.6 to 1.5)
EQ-5D-3L, utility score	0.78 (0.19), 10	0.74 (0.28), 10	0.81(0.10), 8	0.75 (0.17), 10	0.77 (0.18), 8	0.67 (0.35), 9	0.07 (-0.08 to 0.22)
CBQ-HF, Physical	4.5 (5.9), 10	3.7 (4.7), 10	2.0 (4.1), 8	6.3 (6.0), 10	4.4 (7.3) 8	5.2 (5.8), 9	-1.5 (-4.1 to 1.1)

For peer review only - http://bmjopen.bmj.com/site/about/guidelines.xhtml

CBQ-HF, Emotional	17.4 (13.8), 10	18.8 (13.0), 10	15.1 (13.3), 8	20.3 (12.0), 10	15.4 (16.0, 8	22.3 (13.1), 9	-5.1 (-12.5 to 2.3)
CBQ-HF, Social Life	0.7 (1.2), 10	1.6 (2.0), 10	0.4 (0.7), 8	1.8 (2.1), 10	0.6 (1.1), 8	2.2 (2.5), 9	-0.8 (- 2.6 to 1.1)
CBQ-HF, Lifestyle	1.9 (2.3), 10	4.3 (3.2), 10	2.5 (3.1), 8	4.4 (3.3), 10	2.4 (3.2), 8	6.0 (4.5), 9	-1.4 (-4.7 to 1.9)
CC-SCHFI, Maintenance	22.0 (11.0), 10	30.3 (15.7), 10	34.2 (25.1), 8	31.7 (14.6), 10	36.3 (23.5), 8	40.7 (17.9), 9	1.5 (-19.1 to 22.2)
CC-SCHFI, Management	29.0 (21.6), 10	35.6 (14.7), 8	39.3 (28.2), 7	35.0 (18.5), 7	45.0 (13.2), 3	35.0 (19.1), 8	7.4 (-21.4 to 36.2)
CC-SCHFI, Confidence	33.9 (15.6), 10	29.6 (19.8), 10	35.4 (17.6), 8	20.0 (17.2), 10	38.2 (16.4), 8	33.3 (20.6), 9	2.6 (-16.0 to 21.2)

[1] Mean between group differences (intervention minus control) adjusted for baseline values.

For peer review only - http://bmjopen.bmj.com/site/about/guidelines.xhtml

For peer review only

Healthcare utilisation and intervention costs

The average cost of the REACH-HF intervention per patient was estimated to be £362.61. The intervention cost breakdown is provided in Table 2. The wider healthcare and societal utilisation and costs for intervention and control groups are summarised in eTable 7.

Discussion

The findings of this pilot study support the feasibility and acceptability of the home-based REACH-HF rehabilitation intervention in patients with HFpEF and their caregivers, and indicate that it is feasible to recruit and retain participants in a randomised trial of 6-months follow up. The intervention was well received by patients, caregivers, and healthcare facilitators and intervention adherence was good. At follow up, compared to control, a number of patient outcomes showed a potential direction of effect in favour of the intervention group, including our proposed primary outcome of disease-specific HRQoL - MLWHF. We also saw potentially favorable impacts of the REACH-HF intervention on caregiver mental health and measures of burden.

The promising results of this study support the emerging evidence of the impact of exercise-based CR interventions in HFpEF.[6,7] A recent meta-analysis of randomised trials (ranging in sample size from 25 to 198 patients) suggest improvements in exercise capacity and HRQoL following intervention compared with control.[7] However, these previous studies have predominantly been supervised and delivered in centre-based settings. Participation in centre-based CR has been sub-optimal, with national practice surveys indicating that fewer than 20% of eligible HF patients may be receiving exercise-based CR.[8] Therefore, there is increasing interest in home-based programmes that have the potential to overcome these suboptimal rates of CR participation seen with HF.[10,11] To our knowledge, REACH-HF is the first comprehensive self-management CR intervention for HFpEF patients and their caregivers that is home-based, with facilitation by healthcare professionals and whose development is informed by evidence, theory, and input from service users – patients and clinicians.

The mechanism by which CR improves HRQoL in HFpEF remains unclear.[32] Whilst exercise training has been shown to improve cardiac (systolic and diastolic) function in HFrEF patients, studies have failed to show such consistent benefits in HFpEF.[6,7]Instead exercise training may improve exercise tolerance in HFpEF through peripheral mechanisms leading to an improved oxygen extraction in the active skeletal muscles.[33] Such improvements are likely to improve patient physical capacity and hence the physical component of HRQoL. Poor mental health, including depression in

For peer review only - http://bmjopen.bmj.com/site/about/guidelines.xhtml

HF patients is common and may be under recognised and undertreated in cardiac populations such as HFpEF. This is supported by the baseline HADS scores in this study indicating mild to moderate symptoms of depression and anxiety in a proportion of patients (and caregivers). A recent Cochrane review has shown comprehensive CR, including elements of stress management and exercise training, can have significant positive effects in terms in reductions in depression and anxiety of myocardial infarction and post-revascularisation populations.[34] The observed trend towards a reduction depression and anxiety scores with the REACH-HF intervention, points toward a possible basis of improvement in the mental component of HRQoL.

This study has a number of limitations. Firstly, the study was not designed or powered to definitively assess the efficacy or safety of the REACH-HF intervention in HFpEF. Secondly, generalisability of this study's findings is limited, given it was based in a single centre. Thirdly, there was evidence of imbalances between and intervention and control groups in their demographic characteristics and outcome scores at baseline. Fourthly, patient and clinician blinding was not possible in this study because of the nature of the intervention, although we did demonstrate that it was possible to blind outcome assessors to group allocation. Finally, the open label design of the study may have resulted in improvements in patient-reported outcomes in intervention participants as the result of placebo effects. However, we would note there was some evidence of fewer clinical events in the intervention group at 6 months. Given these limitations and the pilot nature of this trial, our findings should therefore be considered preliminary, and encouraging trends require confirmation in a larger, adequately powered clinical trial.

Implications for planning a future trial

Based on MLWHFQ total score as the primary outcome, a full trial comparing the REACH-HF plus usual versus usual care alone would require recruitment of 210 HFpEF patients per group. This estimate is based on detecting a minimum clinically important difference on the MLWHFQ of 5 points,[20] a standard deviation of 25 points (as seen in this pilot trial, see Table 1), a within patient correlation of 0.8 (between baseline and 6-month follow-up calculated from data from this pilot), and an assumed attrition rate of 10% (as seen in this pilot trial, see Figure 1), at 90% power and 5% alpha level.

Two issues raised in this pilot that deserve consideration for a full trial include the choice of exercise test and the assessment of patient adherence to the REACH-HF intervention. In interviews, a number of patients in this study expressed the opinion that they found undertaking the ISWT as an unpleasant experience: 12 of 45 (27%)

For peer review only - http://bmjopen.bmj.com/site/about/guidelines.xhtml

patients were not able to undertake the ISWT at 6-month follow-up. This loss to follow up my have resulted in bias in our assessment of exercise capacity over time and in our comparison of groups. Assessing and ensuring adequate levels of intervention adherence is a challenge in self-directed home based interventions, such as REACH-HF.[11] Levels of patient attendance at face-to-face or telephone contacts with healthcare facilitators indicated good levels of intervention adherence. Patients were also asked to document changes in their health behaviours in a 'Patient Tracker' diary over the duration of the study. We need to examine if these diaries support our conclusion of good intervention adherence seen from facilitator contacts. It will be important to revisit these two issues in the design and planning of a future full trial.

In summary, the findings from this pilot study indicate that the REACH-HF home-based comprehensive self-management CR intervention facilitated by healthcare professional is feasible, acceptable and suggests promising effects on HFpEF patient and caregiver outcomes. This pilot study will help inform the funding application for a fully powered multi-centre randomised trial to assess the clinical effectiveness and cost-effectiveness of the novel REACH-HF intervention in HFpEF patients and their caregivers.

view only

For peer review only - http://bmjopen.bmj.com/site/about/guidelines.xhtml

Acknowledgements

We would like to thank the research nurses (Lynn Rutherford and Fatima Baig) intervention facilitators (Anona Cranston and Gillian Smith) and patients and their caregivers in Dundee and Tayside who participated in this study. We also wish to acknowledge: the data team at Peninsula Clinical Trials Unit (University of Plymouth, UK), Lisa Price (University of Exeter, UK) who assisted with the analysis of accelerometry data, Karen Coyle (University of Dundee) who assisted with qualitative interviews and intervention fidelity checking, and Louise Taylor and her team at the Heart Manual Office Heart Office (Astley Ainslie Hospital, Edinburgh) for their assistance with the preparation and development of the REACH-HF intervention.

Conflicts of interest

RST is the lead for the ongoing portfolio of Cochrane reviews of cardiac rehabilitation. RST and HMD are named Scientific Advisors for ongoing the National Institute of Health and Care Excellence (NICE) updated clinical guidelines for the management heart failure (CG108). HD is an ordinary member of the British Association for Cardiovascular Prevention and Rehabilitation (BACPR) council. All other co-authors declare no conflict of interest to declare.

Funding

This paper presents independent research funded by the National Institute for Health Research (NIHR) under its Programme Grants for Applied Research Programme (Grant Reference Number RP-PG-1210-12004). NB, CA, CJG and RST are also supported by the National Institute for Health Research (NIHR) Collaboration for Leadership in Applied Health Research and Care (CLAHRC) South West Peninsula at the Royal Devon and Exeter NHS Foundation Trust; KJ by CLAHRC West Midlands and SS by CLAHRC East-Midlands. The views expressed are those of the authors and not necessarily those of the NHS, the NIHR or the Department of Health.

For peer review only - http://bmjopen.bmj.com/site/about/guidelines.xhtml

PERSPECTIVES

COMPETENCY IN MEDICAL KNOWLEDGE

The present findings support that patients with HFpEF have a substantial burden with exercise intolerance and a poor health related quality of life

For peer review only

References

1. Dunlay SM, Roger VL, Redfield MM. Epidemiology of heart failure with preserved ejection fraction. *Nat Rev Cardiol.* 2017 May 11.
2. Lam CSP, Donal E, Kraigher-Krainer E, et al Epidemiology and clinical course of heart failure with preserved ejection fraction. *Eur J Heart Fail.* 2011;13:18-28.
3. Liao L, Anstrom KJ, Gottdiener JS, et al Long-term costs and resource use in elderly participants with congestive heart failure in the Cardiovascular Health Study. *Am Heart J.* 2007;153:245-52.
4. Holland DJ, Kumbhani DJ, Ahmed SH, et al Effects of treatment on exercise tolerance, cardiac function, and mortality in heart failure with preserved ejection fraction. A meta-analysis. *J Am Coll Cardiol.* 2011;57:1676-86.
5. Komajda M, Isnard R, Cohen-Solal A, et al. Effect of ivabradine in patients with heart failure with preserved ejection fraction: the EDIFY randomized placebo-controlled trial. *Eur J Heart Fail.* 2017 Apr 30. doi: 10.1002/ejhf.876. [Epub ahead of print]
6. Taylor RS, Davies EJ, Dalal HM, et al Effects of exercise training for heart failure with preserved ejection fraction: a systematic review and meta-analysis of comparative studies. *Int J Cardiol.* 2012;162:6-13.
7. Chan E, Giallauria F, Vigorito C, et al Exercise training in heart failure patients with preserved ejection fraction: a systematic review and meta-analysis. *Monaldi Arch Chest Dis.* 2016;86:759.
8. Dalal HM, Wingham J, Palmer J, et al Why do so few patients with heart failure participate in cardiac rehabilitation? A cross-sectional survey from England, Wales and Northern Ireland. *BMJ Open.* 2012;2:e000787.
9. Beswick AD, Rees K, Griebsch I, et al Provision, uptake and cost of cardiac rehabilitation programmes: improving services to under-represented groups. *Health Technol Assess.* 2004;8(41):iii-iv, ix-x, 1-152.
10. Dalal H, Taylor R. Telehealth technologies could improve suboptimal rates of participation in cardiac rehabilitation. *Heart.* 2016;102:1155-6.
11. Dalal HM, Zawada A, Jolly K, et al Home based versus centre based cardiac rehabilitation: Cochrane systematic review and meta-analysis. *BMJ.* 2010;340.
12. Buckingham SA, Taylor RD, Jolly K, et al Home-based versus centre-based cardiac rehabilitation: abridged Cochrane systematic review and meta-analysis. *Open Heart.* 2016;3(2).
13. Taylor RS, Hayward C, Eyre V, et al Clinical effectiveness and cost-effectiveness of the Rehabilitation Enablement in Chronic Heart Failure (REACH-HF) facilitated self-care rehabilitation intervention in heart failure

For peer review only - http://bmjopen.bmj.com/site/about/guidelines.xhtml

patients and caregivers: rationale and protocol for a multicentre randomised controlled trial. *BMJ Open.* 2015;5(12):e009994.

14. Eyre V, Lang CC, Smith K, et al   Rehabilitation Enablement in Chronic Heart Failure—a facilitated self-care rehabilitation intervention in patients with heart failure with preserved ejection fraction (REACH-HFpEF) and their caregivers: rationale and protocol for a single-centre pilot randomised controlled trial. *BMJ Open*. 2016;6(10).
15. Eldridge SM, Chan CL, Campbell MJ, et al   CONSORT 2010 statement: extension to randomised pilot and feasibility trials. *BMJ.* 2016;355.
16. Piepoli MF, Conraads V, Corra U, et al   Exercise training in heart failure: from theory to practice. A consensus document of the Heart Failure Association and the European Association for Cardiovascular Prevention and Rehabilitation. *Eur J Heart Fail.* 2011;13:347-57.
17. Greaves CJ, Wingham J, Deighan C, et al Optimising self-care support for people with heart failure and their caregivers: development of the Rehabilitation Enablement in Chronic Heart Failure (REACH-HF) intervention using intervention mapping. *Pilot Feasibility Studies*. 2016;2:37.
18. McMurray JJ, Adamopoulos S, Anker SD, et al ESC guidelines for the diagnosis and treatment of acute and chronic heart failure 2012: the task force for the diagnosis and treatment of acute and chronic heart failure 2012 of the European Society of Cardiology. Developed in collaboration with the Heart Failure Association (HFA) of the ESC. *Eur Heart J.* 2012;33.
19. NICE. *Chronic heart failure: Management of chronic heart failure in adults in primary and secondary care*. [Available from: https://www.nice.org.uk/guidance/cg108]
20. Rector T, Kubo S, Cohn J. Patients' self-assessment of their congestive heart failure. Part 2: Content, reliability and validity of a new measure, the Minnesota Living with Heart Failure questionnaire. *Heart Failure.* 1987;Oct/Nov:198-209.
21. Oldridge N, Hofer S, McGee H, et al   The HeartQoL: part II. Validation of a new core health-related quality of life questionnaire for patients with ischemic heart disease. *Eur J Prev Cardiol.* 2014;21:98-106.
22. Pulz C, Diniz RV, Alves AN, et al   Incremental shuttle and six-minute walking tests in the assessment of functional capacity in chronic heart failure. *Can J Cardiol.* 2008;24:131-5.
23. van den Berg-Emons HJ, Bussmann JB, Balk AH, et al   Validity of ambulatory accelerometry to quantify physical activity in heart failure. *Scand J Rehabil Med.* 2000;32:187-92.

For peer review only

For peer review only - http://bmjopen.bmj.com/site/about/guidelines.xhtml

24. Zigmond AS, Snaith RP. The hospital anxiety and depression scale. *Acta Psychiatr Scand.* 1983;67:361-70.
25. Devlin N, Shah K, Feng Y, et al *Valuing Health-Related Quality of Life: An EQ-5D-5L Value Set for England.* Office for Health Economics; 2016. Contract No.: 16/01.
26. Riegel B, Lee CS, Dickson VV, et al An Update on the Self-Care of Heart Failure Index. *J Cardiovasc Nurs.* 2009;24:485-97.
27. Humphrey L, Kulich K, Deschaseaux C, et al The Caregiver Burden Questionnaire for Heart Failure (CBQ-HF): face and content validity. *Health Qual Life Outcomes.* 2013;11:84.
28. Nauser JA, Bakas T, Welch JL. A New instrument to measure quality of life of heart failure family caregivers. *J Cardiovasc Nurs.* 2011;26
29. Curtis L, Burns A. *Unit Costs of Health & Social Care. Personal Services Research Unit.* [Available from: http://www.pssru.ac.uk/project-pages/unit-costs/2016/]
30. van Hout B, Janssen MF, Feng YS, et al Interim scoring for the EQ-5D-5L: mapping the EQ-5D-5L to EQ-5D-3L value sets. *Value in Health.* 2012;15:708-15.
31. Leavy P. *The Oxford handbook of qualitative research*: Oxford University Press; 2014.
32. Nolte K, Herrmann-Lingen C, Wachter R, et al Effects of exercise training on different quality of life dimensions in heart failure with preserved ejection fraction: the Ex-DHF-P trial. *Eur J Prev Cardiol.* 2015;22:582-93. 13.
33. Kitzman DW, Brubaker PH, Herrington DM, et al Effect of endurance exercise training on endothelial function and arterial stiffness in older patients with heart failure and preserved ejection fraction: a randomized, controlled, single-blind trial. *J Am Coll Cardiol*. 2013;62:584–592.
34. Richards SH, Anderson L, Jenkinson CE, et al Psychological interventions for coronary heart disease. *Cochrane Database Syst Rev.* 2017;4:CD002902

Data Sharing

The authors confirm that all data underlying the findings are fully available without restriction. The authors have made the clinical and economic data set available through the University of Exeter's Institutional Repository – Open Research Exeter (see https://ore.exeter.ac.uk). Access to these data is permitted but controlled through requests made via the repository to the chief investigator (Professor Taylor: r.s.taylor @exeter.ac.uk). Although use is permitted, this will be on the basis that the source of

For peer review only

For peer review only - http://bmjopen.bmj.com/site/about/guidelines.xhtml

the data is acknowledged (including the funder) and it includes reference to the data set 'handle'.

FIGURE LEGENDS

Figure 1. CONSORT flow chart

Figure 2. Minnesota Living With Heart failure (MLWHF) outcomes at baseline and at 4 and 6 months follow-up

For peer review only

Contribution Statements

The REACH-HFpEF pilot trial was designed by CCL, KS, HMD, RST, JW, KJ, RCD, PD, JM, RVL, SS (Sally Singh), CA, NB, CJG (Colin Greaves), CG (Colin Green), KP, MH, SS (Susannah Sadler) and CH

KJ, RST, RCD and HMD developed the original idea for REACH-HFpEF

FCW, and CG (Colin Green) did data analysis

VE and CH were responsible for study and data collection management

CCL undertook the first draft of the manuscript that was then edited by JW, FCW, RST and HMD

All authors provided critical evaluation and revision of the manuscript and have given final approval of the manuscript accepting responsibility for all aspects.

For peer review only - http://bmjopen.bmj.com/site/about/guidelines.xhtml

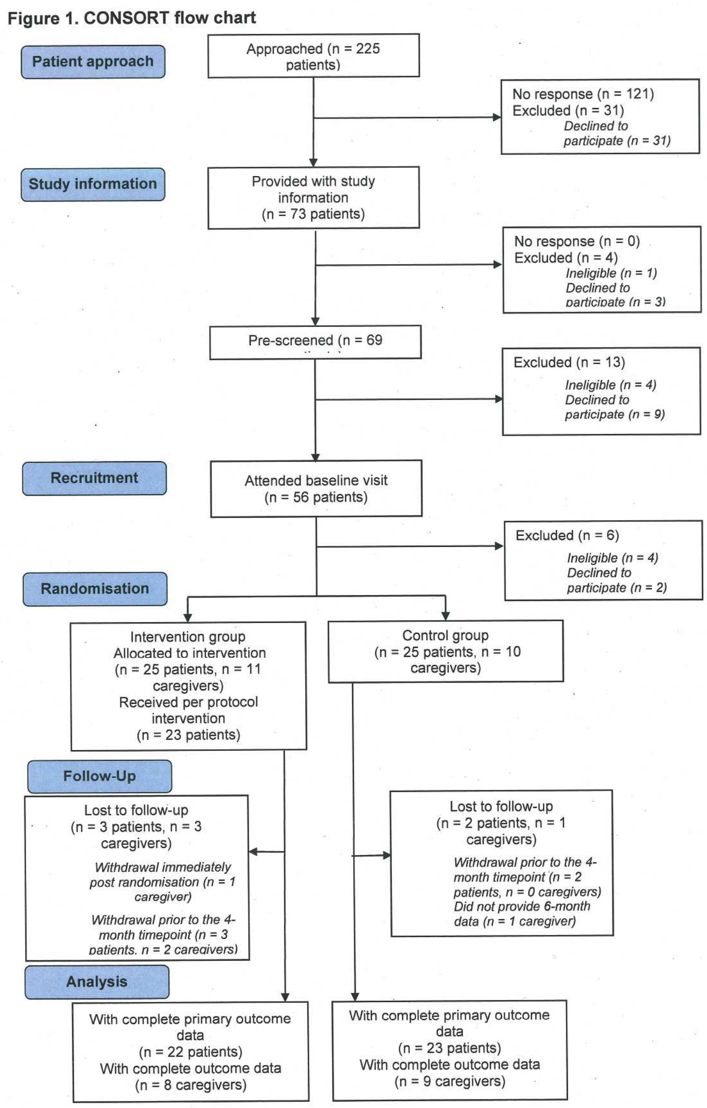

Figure 1. CONSORT flow chart

296x419mm (300 x 300 DPI)

Figure 2.

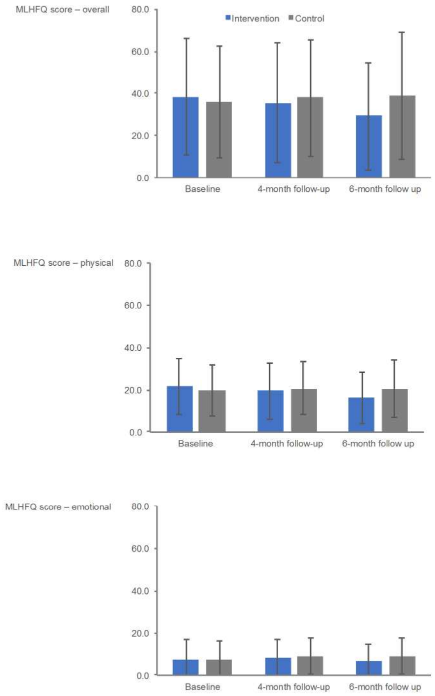

Figure 2. Minnesota Living With Heart Failure (MLWHF) outcomes at baseline and at 4 and 6 month follow-up.

215x279mm (300 x 300 DPI)

eTable1. Unit costs

Resource use/Item	Unit cost 2016 £	Source
**Primary Care cost per visit/appointment**		
GP (surgery)	£31.00	Curtis and Burns, 2016
GP (home)	£74.98	Curtis and Burns, 2015
GP (phone)	£22.29	Curtis and Burns, 2015
Practice nurse (surgery)	£11.11	Curtis and Burns, 2016, Curtis and Burns, 2015
Practice nurse (home)	£18.80	Curtis and Burns, 2016, Curtis and Burns, 2015, Curtis, 2010.
Practice nurse (phone)	£4.30	Curtis and Burns, 2016, Curtis and Burns, 2015
Heart failure nurse	£22.11	Curtis and Burns, 2016,
Physiotherapist	£77.52	Curtis and Burns, 2016, Curtis, 2010.
Occupational therapist	£71.40	Curtis and Burns, 2016, Curtis, 2010.
Community/district nurse	£39.51	Curtis and Burns, 2016, Curtis, 2010.
Health visitor	£27.22	Curtis and Burns, 2015, Curtis, 2010.
Other primary/community service	£22.11	Curtis and Burns, 2016,
**Secondary care cost per event**		
Hospital admission (HF)	£4,668.66	Department of Health, 2016
Hospital admission (non-HF)	£3,966.57	Zannad *et al.* 2011, Department of Health, 2016
Hospital admission (overall)	£4,282.51	Combination of HF and non-HF admission cost, weighted according to admissions recorded in pilot
A&E attendance	£137.82	Department of Health, 2016
Day hospital attendance	£319.33	Department of Health, 2016
Outpatient cardiology appointment	£135.68	Department of Health, 2016
Outpatient cardiac or HF nurse	£102.96	Department of Health, 2016
Other outpatient appointment	£116.54	Department of Health, 2016

For peer review only - http://bmjopen.bmj.com/site/about/guidelines.xhtml

**Social & community care visits:**		
Social worker	£79.00	Curtis and Burns, 2016
Home care /home help	£12.00	Curtis and Burns, 2016
Voluntary agency	£10.00	Curtis and Burns, 2016
Day care	£46.00	Curtis and Burns, 2016
Drop in club	£13.00	Curtis and Burns, 2016
**Medications** (estimated 6-month cost per person)		
Angiotension 2-receptor antagonist	£15.09	Joint Formulary Committee 2017, OpenPrescribing.net
ACE inhibitor	£6.92	Joint Formulary Committee 2017, OpenPrescribing.net
Aldosterone receptor antagonist	£63.05	Joint Formulary Committee 2017, OpenPrescribing.net
Anti-coagulant	£8.34	Joint Formulary Committee 2017, OpenPrescribing.net
Beta-blocker	£6.15	Joint Formulary Committee 2017, OpenPrescribing.net
Digoxin	£18.00	Joint Formulary Committee 2017, OpenPrescribing.net
Ivabradine	£258.24	Joint Formulary Committee 2017, OpenPrescribing.net
Loop diuretic	£7.96	Joint Formulary Committee 2017, OpenPrescribing.net
Nitrate + hydralazine	£589.60	Joint Formulary Committee 2017, OpenPrescribing.net
Thiazide diuretic	£9.61	Joint Formulary Committee 2017, OpenPrescribing.net
**Patient & Caregiver Time** cost per unit		
Caregiver time, hour	£24.00	Curtis and Burns, 2016
Non-caregiver time, hour	£24.00	Curtis and Burns, 2016
Caregiver time off work, per day	£122.31	HM Revenue & Customs, 2017
Non-caregiver time off work, per day	£122.31	
Patient time off work, per day	£96.15	HM Revenue & Customs, 2017

For peer review only - http://bmjopen.bmj.com/site/about/guidelines.xhtml

References

Curtis L, Burns A. *Unit Costs of Health & Social Care 2016*. Personal Social Services Research Unit, The University of Kent. 2016.

Curtis L, Burns A. *Unit Costs of Health & Social Care 2015*. Personal Social Services Research Unit, The University of Kent. 2015.

Curtis L. *Unit Costs of Health & Social Care 2010*. Personal Social Services Research Unit, The University of Kent. 2010.

Department of Health, *NHS reference costs* 2015 to 2016, 2016.

HM Revenue & Customs, *Distribution of median and mean income and tax by age range and gender*: 2014 to 2015, UK National Statistics. 2017

BNF. Joint Formulary Committee. *British National Formulary* (online) London: BMJ Group and Pharmaceutical Press <http://www.medicinescomplete.com> [Accessed on 22.06.2017]

For peer review only

eTable 2. Patient and caregiver acceptability with trial participation at 6-months follow up

**What was your overall impression of taking part in the study?**	**Very good or good** N (%)	**Acceptable** N (%)	**Poor or very Poor** N (%)
**Patients**			
Intervention group, N = 21	19 (90)	2 (10)	0 (0)
Control group, N = 23	23 (100)	0 (0)	0 (0)
Both groups, N =44	42 (95)	2 (5)	0 (0)
**Caregivers**			
Intervention group, N = 8	7 (88)	1 (12)	0 (0)
Control group, N = 9	5 (55)	4 (45)	0 (0)
Both groups, N = 17	13 (72)	5 (28)	0 (0)

For peer review only

For peer review only - http://bmjopen.bmj.com/site/about/guidelines.xhtml

eTable 3. Patients and caregivers acceptability of REACH-HF intervention

The following are verbatim quotes of the positive experiences of the REACH-HF from patients and their caregivers.

Patient: *"I felt like giving her [the REACH-HF intervention facilitator] a hug to say thanks…you don't know what you've done for me… reach doesn't know what they have done for me" …. "Yeh so if anyone is listening to this and I hope 'youse' are and you are wanting to go on this programme, please go on it!!"*

Exercise

Caregiver: *"Yes, it was very helpful [exercise programme]. It really was. Helpful for me, as I say cos I started going out walking. We did the exercises…I'd never seen (my husband) and I laugh so much doing the exercises. You know, we had great fun. And the lady's straight face and…he would…he would put on funny poses and we laughed and we laughed. We thought…you know, we haven't laughed like that for a long, long time, you know. And it was really good. It really was."*

Patient: *After I think it was 9 weeks every single day I was trying my damdest to get past this, but I could not get past the (chair based exercise) warm up thing, so I said the facilitator I'm going to have to stop this (exercise) …. And she went 'no if you can't do that what do you love doing?' I says I love walking so she said 'right if you want to go out for a walk lets go out for a walk'…'*

Role of facilitator (education, support and reassurance)

Patient: *"I think that…reading the manual, talking to 'the nurse ', was very helpful for me in so many different ways. Helping me to understand heart failure….she encouraged me to go out walking…. Just the reassurance that things were better, that there was somebody there that was willing to, erm, say, well, okay, you're doing well. Even just the smallest amount of encouragement. And 'my husband' always felt better after the facilitator went away. Because she felt…almost like a little security blanket, if you want to say. That somebody was there, somebody was asking."*

Facilitator as motivator

For peer review only - http://bmjopen.bmj.com/site/about/guidelines.xhtml

Patient: *'she was wonderful, encouraged me to do more walking and so on and I knew I could do it '*

Supporting behavioural change

Patient: *I tried to watch what I am eating more, my diet I take far more care …. I'm eating a lot more fish and vegetables rather than meat .*

Emotional support for patents and caregivers

Patient: "I'd pulled myself in I was really very inward and they were all saying you should go out with your friends ,or do this, or have them up…..I think being able to speak about it was helpful because that's not me."

Caregiver: *"What I've found about this Programme was….the nurse that came. You could talk it through. After talking to her, I didn't have quite so bad a fear of it [heart failure]. You could tell her how frightened you were,..it's nice to have someone professional to say, well, look, okay, that's that day. I didn't actually realise that until she came, how good it was to actually sit and openly speak about it and openly say, well, ask advice and things. It was lovely having her. You know, it was just a support."*

Caregiver: "I think maybe it's helped him think I can live with this … you know it's not – it doesn't mean the end of things"

For peer review only - http://bmjopen.bmj.com/site/about/guidelines.xhtml

eTable 4. Fidelity of intervention delivery

	Item 1 Involve-ment	Item 2 Assess-ment	Item 3 Plan	Item 4 Under-stand	Item 5a Support – why to change	Item 5b Review	Item 6 Physical activity	Item 7 Emotion	Item 8 Medic-ation	Item 9 Care-giver	Item 10 Care-giver emotion	Item 11 Care-giver well-being	Item 12 Closure
**N patients**	6	6	6	6	6	6	6	6	6	6	6	6	6
**Mean score**	3.3	4.0	3.7	4.5	3.2	3.4	4.5	5.5	5.0	2.5	4.5	2.1	4.0

For peer review only - http://bmjopen.bmj.com/site/about/guidelines.xhtml

eTable 5. Within group difference in patient outcomes between baseline, and 4- and 6-month follow-up

	4-month follow-up vs. baseline Within group mean difference (95% CI)		6-month follow-up vs. baseline Within group mean difference (95% CI)	
	**Intervention**	**Control**	**Intervention**	**Control**
Primary outcome				
MLHFQ, Overall	-2.0 (-9.2 to 5.2)	3.0 (-4.7 to 10.7)	-8.3 (-16.8 to 0.1)	3.9 (-4.9 to 12.6)
MLHFQ, Physical	-1.1 (-4.5 to 2.3)	2.0 (-1.8 to 5.9)	-3.3 (-7.3 to 0.7)	1.6 (-2.7 to 5.9)
MLHFQ, Emotional	-0.6 (-2.7 to 1.5)	1.5 (-1.1 to 4.1)	-1.6 (-3.9to 0.7)	1.3 (-1.5 to 4.2)
Secondary outcomes				
HADS, Anxiety	0.0 (-1.4 to 1.3)	0.7 (-0.8 to 2.1)	0.1 (-1.5 to 1.8)	0.3 (-1.6 to 2.2)
HADS, Depression	-0.4 (-1.7 to 0.8)	1.0 (0.1 to 2.0)	-0.2 (-1.2 to 0.7)	1.3 (-0.2 to 2.8)
Heart-QoL, Global	0.0 (-0.2 to 0.3)	-0.2 (-0.5 to 0.0)	0.3 (0.0 to 0.5)	-0.2 (-0.6 to 0.1)
Heart-QoL, Physical	0.1 (-0.2 to 0.3)	-0.2 (-0.5 to 0.0)	0.3 (0.0 to 0.6)	-0.2 (-0.6 to 0.1)
Heart-QoL, Emotional	0.0 (-0.3 to 0.2)	-0.2 (-0.5 to 0.2)	0.1 (-0.1 to 0.4)	-0.2 (-0.6 to 0.2)
EQ-5D-3L, utility score	0.01 (-0.1 to 0.12)	-0.06 (-0.12 to -0.01)	0.05 (-0.08 to 0.18)	-0.03 (-0.12 to 0.07)
SCHFI, Maintenance	15.5 (9.4 to 21.5)	5.8 (1.1 to 10.6)	9.8 (4.5 to 14.8)	5.1 (-1.5 to 11.8)
SCHFI, Management	12.1 (1.3 to 22.9)	-5.4 (-14.9 to 4.2)	8.6 (-4.4 to 21.6)	-1.0 (-14.5 to 12.5)
SCHFI, Confidence	3.5 (-10.0 to 17.0)	-7.0 (-15.4 to 1.4)	0.2 (-10.4 to 10.8)	-3.1 (-15.3 to 9.0)
ISWT (metres)	5.0 (-27.9 to 37.9)	-12.9 (-41.3 to 15.4)	-7.9 (-44.6 to 28.7)	4.1 (-17.3 to 25.5)
Accelerometry, average time/day at ≤ 20mg	-9 (-36 to 18)	26 (5 to 48)	8 (-14 to 30)	26 (-5 to 60)

For peer review only

For peer review only - http://bmjopen.bmj.com/site/about/guidelines.xhtml

Accelerometry, average time/day at 21 to 40mg	11 (1 to 20)	-13 (-22 to -3)	5 (-3 to 13)	-11 (-25 to 2)
Accelerometry, average time/day at 41 to 60mg	2 (-6 to 10)	-6 (-12 to 0)	-2 (-8 to 5)	-7 (-17 to 3)
Accelerometry, average time/day at 61 to 80mg	0 (-5 to 6)	-3 (-7 to 1)	-4 (-10 to 2)	-4 (-10 to 2)
Accelerometry, average time/day at 81 to 100mg	0 (-3 to 3)	-1 (-4 to 1)	-2 (-5 to 1)	-1 (-5 to 2)
Accelerometry, average time/day at > 100mg	-4 (-9 to 1)	-3 (-6 to 0)	-5 (-11 to 1)	-4 (-8 to 1)

For peer review only

For peer review only - http://bmjopen.bmj.com/site/about/guidelines.xhtml

eTable 6. Within group difference in caregiver outcomes between baseline, and 4- and 6-month follow-up

	4-month follow-up vs. baseline<br>Within group mean difference (95% CI)		6-month follow-up vs. baseline<br>Within group mean difference (95% CI)	
	**Intervention**	**Control**	**Intervention**	**Control**
HADS, Anxiety	-2.1 (-5.4 to 1.1)	0.6 (-2.2 to 3.4)	-3.0 (-5.5 to -0.5)	0.9 (-1.5 to 3.3)
HADS, Depression	0.3 (-2.2 to 2.7)	0.7 (-1.6 to 3.0)	-0.8 (-2.6 to 1.1)	0.9 (-2.2 to 4.0)
FAMQOL, Overall	-3.0 (-10.4 to 4.4)	-2.6 (-6.7 to 1.5)	-6.3 (-13.1 to 0.6)	-1.9 (-7.8 to 3.9)
FAMQOL, Physical	-1.8 (-4.1 to 0.6)	0.0 (-2.3 to 2.3)	-1.9 (-3.3 to -0.4)	0.6 (-0.6 to 1.7)
FAMQOL, Psychological	-0.6 (-3.0 to 1.8)	-1.5 (-3.8 to 0.8)	-1.1 (-3.7 to 1.5)	-1.2 (-4.0 to 1.6)
FAMQOL, Social	-1.4 (-3.7 to 1.0)	-1.0 (-3.5 to 1.5)	-2.0 (-3.4 to -0.6)	-0.6 (-3.1 to 2.0)
EQ5D-3L, utility score	0.01 (-0.05 to 0.07)	0.01 (-0.10 to 0.12)	-0.03 (-0.12 to 0.07)	-0.08 (-0.19 to 0.02)
CBQ-HF, Physical	-2.4 (-5.6 to 0.8)	2.6 (-0.7 to 5.9)	0.0 (-2.4 to 2.4)	1.4 (-0.1 to 3.0)
CBQ-HF, Emotional	-1.1 (-5.0 to 2.8)	1.5 (-3.1 to 6.1)	-0.9 (-5.1 to 3.4)	4.0 (-2.4 to 10.4)
CBQ-HF, Social Life	-0.1 (-0.4 to 0.2)	0.2 (-0.8 to 1.2)	0.1 (-0.4 to 0.7)	0.6 (-1.1 to 2.2)
CBQ-HF, Lifestyle	0.8 (-1.2 to 2.7)	0.1 (-1.5 to 1.7)	0.6 (-1.4 to 2.7)	2.0 (-0.4 to 4.4)
CC-SCHFI, Maintenance	13.8 (-6.0 to 33.5)	1.4 (-5.6 to 8.4)	15.9 (-2.9 to 34.6)	11.9 (0.6 to 23.1)
CC-SCHFI, Management	5.0 (-10.1 to 20.1)	7.5 (-11.5 to 26.5)	5.0 (-27.9 to 37.9)	-1.4 (-19.3 to 16.4)

For peer review only - http://bmjopen.bmj.com/site/about/guidelines.xhtml

CC-SCHFI, Confidence	2.1 (-13.4 to 17.6)	-9.6 (-23.7 to 4.6)	4.9 (-11.1 to 20.8)	5.4 (-10.9 to 21.8)

For peer review only

For peer review only - http://bmjopen.bmj.com/site/about/guidelines.xhtml

Table e7. Wider healthcare and societal utilisation at 6-months follow up

	Intervention		Control	
	Appointments/ visits per person	Cost £ per person	Appointments/ visits per person	Cost £ per person
	mean (SD) N	mean (SD)	mean (SD) N	mean (SD)
**Primary Care Appointments**				
GP (surgery)	5.36 (7.68) 22	£166.16 (£238.08)	2.78 (2.04) 23	£86.18 (£63.24)
GP (home)	0.45 (0.91) 22	£33.74 (£68.24)	0.61 (2.29) 23	£45.74 (£171.71)
GP (phone)	0.64 (1.29) 22	£14.27 (£28.76)	0.91 (3.36) 23	£20.29 (£74.90)
Practice nurse (surgery)	2.77 (2.69) 22	£30.77 (£29.88)	2.61 (2.52) 23	£28.99 (£27.99)
Practice nurse (home)	0.09 (0.43) 22	£1.69 (£8.08)	0.00 (0.00) 23	£0.00 (£0.00)
Practice nurse (phone)	0.27 (0.94) 22	£1.16 (£4.04)	0.39 (1.88) 23	£1.68 (£8.08)
Heart failure nurse	0.00 (0.00) 22	£0.00 (£0.00)	0.00 (0.00) 23	£0.00 (£0.00)
Physiotherapist	2.73 (12.79) 22	£211.62 (£984.45)	1.00 (3.80) 23	£77.52 (£294.56)
Occupational therapist	0.00 (0.00) 22	£0.00 (£0.00)	0.52 (2.50) 23	£39.05 (£187.74)
Community/district nurse	0.05 (0.21) 22	£1.98 (£8.30)	0.39 (1.88) 23	£15.41 (£74.27)
Health visitor	0.00 (0.00) 22	£0.00 (£0.00)	0.00 (0.00) 23	£0.00 (£0.00)
Primary Care Total	12.36 (17.84) 22	£461	9.22 (11.10) 23	£315
**Secondary care**				
Hospital admission	0.18 (0.50) 22	£770.85 (£2141.25)	0.30 (0.63) 23	£1284.75 (£2697.98)
A&E attendance	0.00 (0.00) 22	£0.00 (£0.00)	0.09 (0.29) 23	£12.40 (£39.97)

For peer review only - http://bmjopen.bmj.com/site/about/guidelines.xhtml

Day hospital attendance	0.32 (0.72) 22	£102.18 (£229.92)	0.04 (0.21) 23	£12.77 (£67.06)
Outpatient cardiology appointment	0.41 (0.67) 2	£55.63 (£90.90)	0.57 (1.08) 23	£77.34 (£146.53)
Outpatient cardiac or HF nurse	0.05 (0.21) 22	£5.15 (£21.62)	0.00 (0.00) 23	£0.00 (£0.00)
Other outpatient appointment	0.00 (0.00) 22	£0.00 (£0.00)	0.00 (0.00) 23	£0.00 (£0.00)
Secondary Care Total	0.95 (1.00) 22	£934	1.00 (1.48) 23	£1,387
Social worker	0.45 (1.41) 22	£35.55 (£111.39)	0.00 (0.00) 23	£0.00 (£0.00)
Home care /home help	4.41 (20.68) 22	£52.92 (£247.20)	3.48 (11.01) 23	£41.76 (£132.00)
Day care	0.00 (0.00) 22	£0.00 (£0.00)	6.26 (20.74) 23	£287.96 (£952.20)
Drop in club	0.00 (0.00) 22	£0.00 (£0.00)	0.00 (0.00) 23	£0.00 (£0.00)
Other day care service	0.00 (0.00) 22	£0.00 (£0.00)	0.00 (0.00) 23	£0.00 (£0.00)
Social Care Total	4.86 (20.85) 22	£88	9.74 (22.49) 23	£330
Voluntary agency visit	0.00 (0.00) 22	£0.00 (£0.00)	0.09 (0.42) 23	£0.90 (£4.20)
Other primary or community based service	0.00 (0.00) 22	£0.00 (£0.00)	0.16 (0.80) 23	£3.54 (£17.69)
All Health & Social Care Visits Total	18.18	£1,484	20.20	£2,036
	% prescribed	Cost per person	% prescribed	Cost per person

For peer review only - http://bmjopen.bmj.com/site/about/guidelines.xhtml

	Mean, N	mean	Mean, N	mean
**Medications**				
Angiotensin II receptor antagonist	29% 25	£4.38	28%, 25	£4.23
ACE inhibitor	44%, 25	£3.04	48%, 25	£3.31
Aldosterone receptor antangonist	16%, 25	£10.09	24%, 25	£15.13
Anti-coagulant	15%, 25	£1.25	53%, 25	£4.42
Beta-blocker	56%, 25	£3.43	44%, 25	£2.69
Digoxin	8%., 25	£1.44	12%, 25	£2.16
Ivabradine	4%, 25	£10.33	4%, 25	£10.33
Loop diuretic	77%, 25	£6.14	76%, 25	£6.06
Nitrate	39%, 25	£108.15	19%, 25	£52.69
Thiazide diuretic	5%, 25	£0.48	1%, 25	£0.10
All Medications Total		£149		£101
All Health & Social Care Total		£1,632		£2,137
**Informal care**				
Caregiver hours per week	3.03 (5.86) 22	£72.72 (£140.64)	12.41 (30.30) 23	£297.60 (£727.20)

only

For peer review only - http://bmjopen.bmj.com/site/about/guidelines.xhtml

Non-caregiver hours per week	4.98 (12.57) 22	£119.52 (£301.68)	0.46 (1.31) 23	£11.04 (£31.44)
Total caring hours per week	8.01	£192	12.86	£309
Total caring hours per 6 months	208	£4,998.24	334	£8,025
Caregiver days off work	0.14 (0.64) 22	£17.12 (£78.28)	1.00 (4.38) 23	£122.31 (£535.71)
Non-caregiver days off work	0.14 (0.64) 22	17.12 (£78.28)	0.00 (0.00) 13	£0.00 (£0.00)
Total days off work (6-mths)	0.28	£34.25	1	£122.31
Patient days off work	0.00 (0.00) 22	£0.00 (£0.00)	0.00 (0.00) 23	£0.00 (£0.00)
Informal Care Total		£5,032		£8,147
**All Health, Informal & Social Care Total**		**£6,665**		**£10,284**

For peer review only - http://bmjopen.bmj.com/site/about/guidelines.xhtml

CONSORT 2010 checklist of information to include when reporting a pilot or feasibility trial*

Section/Topic	Item No	Checklist item	Reported on page No
**Title and abstract**			
	1a	Identification as a pilot or feasibility randomised trial in the title	1
	1b	Structured summary of pilot trial design, methods, results, and conclusions (for specific guidance see CONSORT abstract extension for pilot trials)	3
**Introduction**			
Background and objectives	2a	Scientific background and explanation of rationale for future definitive trial, and reasons for randomised pilot trial	4
	2b	Specific objectives or research questions for pilot trial	5
**Methods**			
Trial design	3a	Description of pilot trial design (such as parallel, factorial) including allocation ratio	6
	3b	Important changes to methods after pilot trial commencement (such as eligibility criteria), with reasons	NA
Participants	4a	Eligibility criteria for participants	6
	4b	Settings and locations where the data were collected	6
	4c	How participants were identified and consented	6. Protocol paper (ref 14)
Interventions	5	The interventions for each group with sufficient details to allow replication, including how and when they were actually administered	7, Protocol paper (ref 14)
Outcomes	6a	Completely defined prespecified assessments or measurements to address each pilot trial objective specified in 2b, including how and when they were assessed	8,9
	6b	Any changes to pilot trial assessments or measurements after the pilot trial commenced, with reasons	NA
	6c	If applicable, prespecified criteria used to judge whether, or how, to proceed with future definitive trial	10
Sample size	7a	Rationale for numbers in the pilot trial	Protocol paper (ref 14)
	7b	When applicable, explanation of any interim analyses and stopping guidelines	NA
Randomisation:			
Sequence generation	8a	Method used to generate the random allocation sequence	Protocol paper (ref 14)
	8b	Type of randomisation(s); details of any restriction (such as blocking and block size)	Protocol

For peer review only

			paper (ref 14)
Allocation concealment mechanism	9	Mechanism used to implement the random allocation sequence (such as sequentially numbered containers), describing any steps taken to conceal the sequence until interventions were assigned	Protocol paper (ref 14)
Implementation	10	Who generated the random allocation sequence, who enrolled participants, and who assigned participants to interventions	Protocol paper (ref 14)
Blinding	11a	If done, who was blinded after assignment to interventions (for example, participants, care providers, those assessing outcomes) and how	Protocol paper (ref 14)
	11b	If relevant, description of the similarity of interventions	
Statistical methods	12	Methods used to address each pilot trial objective whether qualitative or quantitative	Protocol paper (ref 14)
**Results**			
Participant flow (a diagram is strongly recommended)	13a	For each group, the numbers of participants who were approached and/or assessed for eligibility, randomly assigned, received intended treatment, and were assessed for each objective	Fig 1
	13b	For each group, losses and exclusions after randomisation, together with reasons	Fig 1
Recruitment	14a	Dates defining the periods of recruitment and follow-up	10
	14b	Why the pilot trial ended or was stopped	NA
Baseline data	15	A table showing baseline demographic and clinical characteristics for each group	11,12,13
Numbers analysed	16	For each objective, number of participants (denominator) included in each analysis. If relevant, these numbers should be by randomised group	13,14,15,16,17,18,19,20,21,22,23,24
Outcomes and estimation	17	For each objective, results including expressions of uncertainty (such as 95% confidence interval) for any estimates. If relevant, these results should be by randomised group	13,14,15,16,17,18,19,20,21,22,23,24
Ancillary analyses	18	Results of any other analyses performed that could be used to inform the future definitive trial	NA
Harms	19	All important harms or unintended effects in each group (for specific guidance see CONSORT for harms)	17
	19a	If relevant, other important unintended consequences	
**Discussion**			
Limitations	20	Pilot trial limitations, addressing sources of potential bias and remaining uncertainty about feasibility	26
Generalisability	21	Generalisability (applicability) of pilot trial methods and findings to future definitive trial and other studies	26,27
Interpretation	22	Interpretation consistent with pilot trial objectives and findings, balancing potential benefits and harms, and considering other relevant evidence	26,27
	22a	Implications for progression from pilot to future definitive trial, including any proposed amendments	26,27

**Other information**			
Registration	23	Registration number for pilot trial and name of trial registry	1
Protocol	24	Where the pilot trial protocol can be accessed, if available	Protocol paper (ref 14)
Funding	25	Sources of funding and other support (such as supply of drugs), role of funders	28
	26	Ethical approval or approval by research review committee, confirmed with reference number	6, Protocol paper (ref 14)

Citation: Eldridge SM, Chan CL, Campbell MJ, Bond CM, Hopewell S, Thabane L, et al. CONSORT 2010 statement: extension to randomised pilot and feasibility trials. BMJ. 2016;355.
*We strongly recommend reading this statement in conjunction with the CONSORT 2010, extension to randomised pilot and feasibility trials, Explanation and Elaboration for important clarifications on all the items. If relevant, we also recommend reading CONSORT extensions for cluster randomised trials, non-inferiority and equivalence trials, non-pharmacological treatments, herbal interventions, and pragmatic trials. Additional extensions are forthcoming: for those and for up to date references relevant to this checklist, see www.consort-statement.org.

For peer review only
